# High‐efficiency genome editing using a *dmc1* promoter‐controlled CRISPR/Cas9 system in maize

**DOI:** 10.1111/pbi.12920

**Published:** 2018-04-30

**Authors:** Chao Feng, Handong Su, Han Bai, Rui Wang, Yalin Liu, Xianrui Guo, Chang Liu, Jing Zhang, Jing Yuan, James A. Birchler, Fangpu Han

**Affiliations:** ^1^ State Key Laboratory of Plant Cell and Chromosome Engineering Institute of Genetics and Developmental Biology Chinese Academy of Sciences Beijing China; ^2^ University of Chinese Academy of Sciences Beijing China; ^3^ Division of Biological Sciences University of Missouri Columbia MO USA

**Keywords:** maize, targeted genome editing, CRISPR/Cas9, *dmc1* promoter

## Abstract

Previous studies revealed that the promoters for driving both *Cas9* and sgRNAs are quite important for efficient genome editing by CRISPR/Cas9 in plants. Here, we report our results of targeted genome editing using the maize *dmc1* gene promoter combined with the *U3* promoter for *Cas9* and sgRNA, respectively. Three loci in the maize genome were selected for targeting. The T0 plants regenerated were highly efficiently edited at the target sites with homozygous or bi‐allelic mutants accounting for about 66%. The mutations in T0 plants could be stably transmitted to the T1 generation, and new mutations could be generated in gametes or zygotes. Whole‐genome resequencing indicated that no off‐target mutations could be detected in the predicted loci with sequence similarity to the targeted site. Our results show that the *dmc1* promoter‐controlled (DPC) CRISPR/Cas9 system is highly efficient in maize and provide further evidence that the optimization of the promoters used for the CRISPR/Cas9 system is important for enhancing the efficiency of targeted genome editing in plants. The evolutionary conservation of the *dmc1* gene suggests its potential for use in other plant species.

## Introduction

The clustered regularly interspaced short palindromic repeat (CRISPR)/CRISPR‐associated 9 (Cas9) system is derived from a bacterial immune system and has been widely used for targeted genome editing (Cong *et al*., 2013; Hsu *et al*., 2014; Mali *et al*., 2013). Unlike the two formerly developed sequence specific nucleases (SSNs), zinc finger nucleases (ZFNs) and transcription activator‐like effector nucleases (TALENs), the CRISPR/Cas9 is an RNA‐guided endonuclease to generate DNA double‐strand breaks (DSBs) at the target sites of the genome (Gaj *et al*., 2013). Usually two pathways, including nonhomologous end‐joining (NHEJ) or homology‐directed repair (HDR), are induced to repair the DSBs. The NHEJ pathway is error‐prone and frequently generates indels at the repair sites, while HDR could be adopted for precise sequence replacement or insertion when a template DNA is provided (Puchta and Fauser, 2014).

In 2013, targeted genome editing by CRISPR/Cas9 was first reported in mammalian cell lines (Cong *et al*., 2013; Mali *et al*., 2013). Later this system has been widely used in various species (DiCarlo *et al*., 2013; Friedland *et al*., 2013; Gratz *et al*., 2013; Hwang *et al*., 2013; Jiang *et al*., 2013; Li *et al*., 2013a,c; Yu *et al*., 2013). In plants, CRISPR/Cas9‐mediated genome editing was first simultaneously reported in three model species (Li *et al*., 2013b; Nekrasov *et al*., 2013; Shan *et al*., 2013). Since then, genome editing in plants has been reported by several other groups (Brooks *et al*., 2014; Feng *et al*., 2013; Liang *et al*., 2014; Miao *et al*., 2013; Xie and Yang, 2013; Xing *et al*., 2014). These reports provide evidence that the CRISPR/Cas9 could be used for targeted genome editing in plants; however, the editing efficiency is relatively low.

To enhance the efficiency of CRISPR/Cas9‐mediated genome editing in plants, improvements need to be made. In vertebrates, the *Cas9* (mRNA or protein) and sgRNAs could be simultaneously injected into the zygotic cells. Bi‐allelic mutations for multiple sites in the genome took place before the first cell division, thus generating multigene bi‐allelic mutants (Friedland *et al*., 2013; Hwang *et al*., 2013; Li *et al*., 2013a,c). In this way, the dose of Cas9 and sgRNAs could be controlled to improve the targeting possibility. However, plant zygotic cells are difficult to be transformed directly due to technological limitations. At present, tissue culture is necessary for transformation of most plant species, which is preferentially mediated by Agrobacterium. Thus, for most plants, callus cells are exposed to gene editing reagents for initial targeting. The targeted editing efficiency in calli cells directly determines the proportion of targeted mutant output. Studies in rice and other species suggest the factors that could influence the targeting efficiency include the codons used for *Cas9* translation, promoters used for both the sgRNAs and *Cas9* gene expression and so on (Ma *et al*., 2015; Xie *et al*., 2015; Xu *et al*., 2014; Zhang *et al*., 2014). For example, the *Ubiquitin* gene promoter apparently works better than the CaMV *35S* promoter in rice. In *Arabidopsis*, the CaMV *35S* promoter used for *Cas9* generated only chimeras in the T1 generation without homozygotes or bi‐allelic mutants (Feng *et al*., 2013, 2014; Xing *et al*., 2014). However, an egg cell‐specific promoter used for *Cas9* could generate triple mutants in the T1 generation (Wang *et al*., 2015).

Maize is one of the most important crops in the world. Traditionally, genetic study of maize depends on chemical mutagenesis using ethyl methane sulphonate (EMS) or transposon tagging to generate mutants. Both are time consuming for mutant screening and the required generations of backcrossing (Nannas and Dawe, 2015). Efficient targeted genome editing in maize could simplify the mutant creation process. Targeted genome editing by CRISPR/Cas9 in maize protoplasts was first reported in 2014 (Liang *et al*., 2014). Thereafter, another group reported the generation of mutants of the *ZmHKT* gene in the maize B73 inbred line (Xing *et al*., 2014). The maize *ubiquitin1* gene promoter and rice *U3* or wheat *U3* promoters were used for a maize codon‐optimized *Cas9* (*zCas9*) and sgRNAs, respectively. The high efficiency of CRISPR/Cas9‐mediated targeted mutation in maize was also reported for three targeted alleles when using biolistic‐mediated transformation in one report (Svitashev *et al*., 2015). In another two reports, the combination of *ubiquitin1* gene promoter and maize *U6* promoter, or CaMV *35S* promoter and maize *U3* promoter for *Cas9* and sgRNAs generated mutations at 13% or 2% in T0 generation seedlings when using Agrobacterium‐mediated *Cas9*/sgRNA delivery; most seedlings are chimeras (Feng *et al*., 2016; Zhu *et al*., 2016). More recently, when using the maize *ubiquitin1* gene promoter and two rice *U6* promoters for a rice codon‐optimized *Cas9* and sgRNAs, mutations generated by a combination of two sgRNAs targeted at one gene were reported up to 70%, with bi‐allelic mutations at a frequency of 22%–58% for four genes (Char *et al*., 2016). Most recently, a high frequency of targeted mutation for one site in the *LG1* gene in maize ZC01 inbred line was reported (Li *et al*., 2017). In most reports, *Cas9* is driven by the maize *ubiquitin1* promoter and indeed proved to be more efficient than CaMV *35S*. However, with the use of different promoters for sgRNA expression, the targeting efficiency varied significantly from each other, although the same promoter was used for *Cas9*. These studies revealed that the mutation efficiency of CRISPR/Cas9 system largely depends on both the expressions of *Cas9* and sgRNAs. Thus, screening and testing of new combinations of promoters for both *Cas9* and sgRNA will improve targeted genome editing efficiency in plant species.

Here, we report our results of using a maize *dmc1* promoter‐controlled (DPC) CRISPR/Cas9 system and generating highly efficient targeted genome editing in maize Hi‐II germplasm. Targeting a locus with one sgRNA, the T0 plants regenerated were high‐efficiently edited at the target sites with homozygous or bi‐allelic mutants accounting for about 66%, with the remainder heterozygous or mosaic mutants. Mutations in the T0 plants could be stably transmitted to the next generation, and at the same time, new mutations would be generated. Whole‐genome resequencing detected no mutations at the predicted off‐target sites.

## Results

### *Dmc1* promoter‐controlled (DPC) CRISPR/Cas9‐mediated targeted editing of *zb7* in maize T0 generation

In our previous work, the *35S* promoter was used for driving the *Cas9* (human codon‐optimized) expression in maize. A screen of over one hundred mutant lines only produced one plant with the mutant phenotype. Most of the regenerated plants were mosaic with a low mutation ratio (Feng *et al*., 2016). In this work, our purpose was to choose a meiosis‐specific promoter for increasing the mutation efficiency and at the same time avoiding the mosaicism. Thus, we chose the promoter of the maize *dmc1* gene, which was reported to be expressed specifically in meiocytes (Klimyuk and Jones, 1997) and reasoned that gametes in T0 plants could be mutated and then recover homozygous or bi‐allelic mutants in the T1 generation at high frequency.

**Figure 1:**
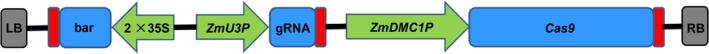
Schematic illustration of DPC CRISPR/Cas9 expression T‐DNA. Maize *dmc1* gene and *U3* promoters were used for Cas9 and sgRNA, respectively.

**Figure 2:**
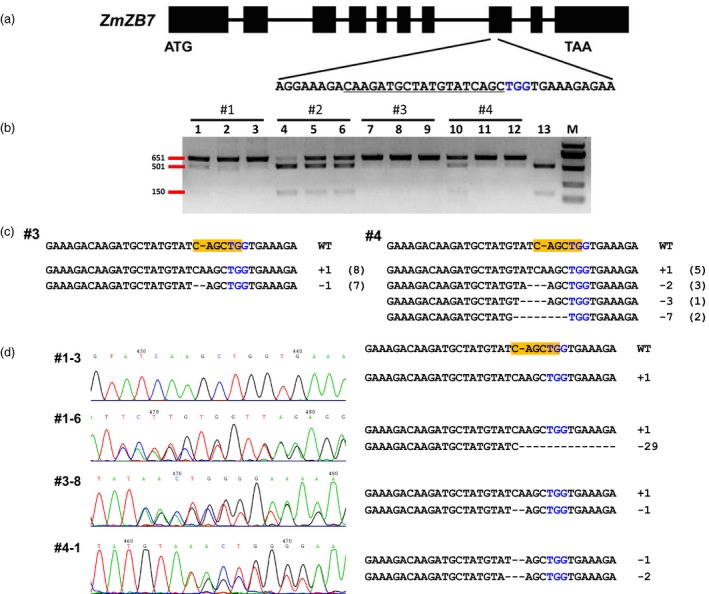
Identification of mutations in the *zb7* gene by RFLP assay and Sanger sequencing. (a) Schematic illustration of the target site in the *zb7* gene. Black box indicates exons, while the lines between them represent introns. Underlined sequence was selected for targeting; nucleotides marked in blue represent PAM (protospacer adjacent motif). (b) RFLP assay of the genomic DNA of four transgene‐positive calli (#1–#4) in the first batch. For each callus, three independent samples were collected and used. Lane 1–3, calli #1; lane 4–6, calli #2; lane 7–9, calli #3; lane 10–12, calli #4; lane 13, control (wild‐type DNA amplicons digested with *Pvu* II). M, DNA marker. Primer pair zb7‐F/zb7‐R was used for PCR amplification, *Pvu* II was used for digestion. (c) Mutation analysis of two transgene‐positive calli by cloning followed by Sanger sequencing. #3, #4 were the two calli sample with homozygous or bi‐allelic mutations by RFLP assay. (d) Sanger sequencing results of mutations in regenerated seedlings. #1–3, #1–6, #3–8, #4–1 were selected seedlings used for analysis. Left, sequencing chromatograph and right, the edited sequences at the target site. The yellow box indicates the *Pvu* II site.

In our DPC CRISPR/Cas9 system, the expression of *Cas9* gene is controlled by the maize *dmc1* gene promoter and *nos* terminator, and the sgRNA is driven by the maize *U3* promoter (Figure 1; Leader *et al*., 1994). We also checked the efficiency of targeted gene editing by *U3* and *U6* promoters in protoplasts (Zhu *et al*., 2016); the results revealed that the maize *U3* promoter works better than *U6* for the sites examined (Figure S1). To make the later analysis simpler, the maize *zb7* gene was chosen as a target gene due to its albino phenotype when completely knocked out (Figure 2a; Lu *et al*., 2012). For targeting the *zb7* gene, the DPC CRISPR/Cas9 constructs were transformed into maize Hi‐II immature embryos by Agrobacterium in two batches, and in total, 10 bialaphos‐resistant calli (transgene‐positive calli) were obtained. Although we supposed that the *dmc1* promoter‐controlled *Cas9* gene should be specifically expressed in reproductive tissue, we checked the expression of the *Cas9* gene in the transgene‐positive calli. Total RNA was extracted from calli of the first batch (three of the four transgene‐positive calli) and was used for expression analysis. To our surprise, the *Cas9* gene had a relatively high expression level in the calli (Figure S2a). To further test whether the *dmc1* promoter is active in calli, we compared the expression level of *dmc1* gene in different tissues. The results indicated that the *dmc1* gene was highly expressed in callus and tassel, but weakly expressed in leaf and root (Figure S2b). These results prompted us to further identify whether the target locus was edited in calli. We then extracted genomic DNA from the four calli of the first batch and for each one we produced three independent sample duplicates. Because there was a restriction site coincident with the Cas9‐cutting site, by RFLP (restriction fragment length polymorphism) assay of the PCR amplicons involving the target site, we were again surprised to find that the *zb7* gene was edited in all four calli, with homozygous or bi‐allelic mutations present in three of them (Figure 2b). In some calli, the mutation types of the three duplicate samples were different and indicated the editing occurred at different stages. In the second batch of transformations, six transgene‐positive calli were obtained, homozygous or bi‐allelic mutations could also be detected. To confirm the mutations in calli revealed by RFLP assays, the PCR products of the target site were directly subjected to Sanger sequencing. The sequencing results showed mono‐allelic, di‐allelic or multi‐allelic mutations in calli, and the mutation types were mostly small insertions and deletions (indels; Figure 2c). The pattern of mutation sequences in calli also indicated that mutations occurred at different stages during calli development.

**Figure 3:**
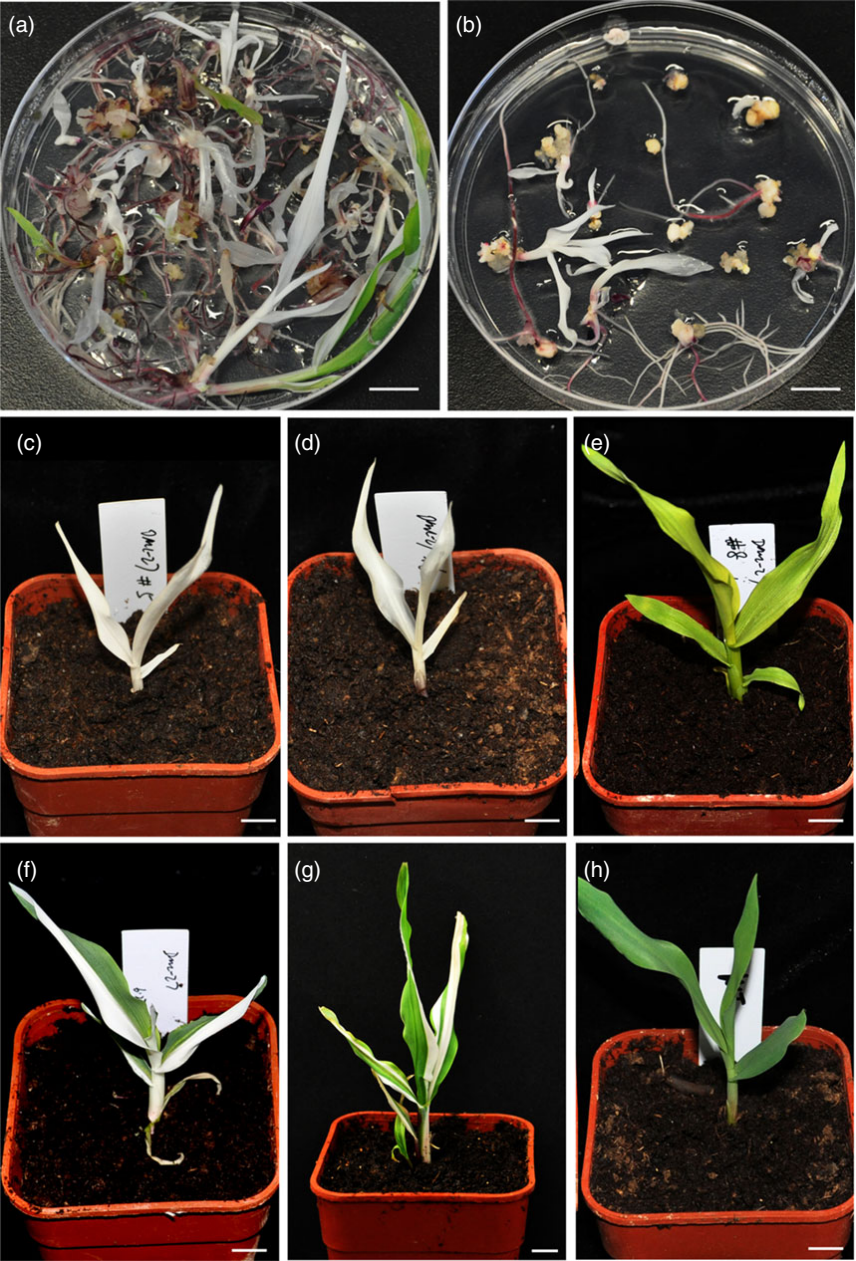
Phenotypes of selected T0 seedlings created by DPC CRISPR/Cas9 at the *zb7* gene. (a, b) Seedling regenerated from two independent transgene‐positive calli. (c–h) Selected seedlings show different representative phenotypes. (c) and (d), albino. (e), chlorosis. (f) and (g), chimera. (h), control (wild‐type Hi‐II seedling). Bars = 1 cm.

The transgene‐positive calli were then cultured for regeneration. For the two batches, in total, 237 seedlings were regenerated from the ten calli. There were four kinds of phenotypes observed among all the seedlings, including albino, chlorosis, chimeric and wild type (Figure 3a–h), with the ratio for each one 51%, 15%, 19% and 15%, respectively (Table S1). The genotyping results indicated that the genotype corresponds to the phenotype. In albino seedlings, the mutations in both the two alleles were found to cause frameshift during translation, thus completely knocking out the gene. In chlorotic seedlings, bi‐allelic mutations were detected with a 3 bp deletion and a 11 bp deletion for the two alleles, respectively. Because the 3 bp deletion only led to a glutamine deletion at the protein level but not a frameshift, this kind of mutated allele may act as a weak allele and still retain partial function (Figure S3a). In fact, the chlorotic seedling could only grow for 3–4 weeks before it perished; the albino seedlings can just survive 2 weeks. In chimeric seedlings, a high ratio of mutant alleles could be detected, and in wild‐type seedlings, mono‐allelic or low‐ratio of mutant alleles was detected. The phenotypes of the seedlings were also consistent with the genotyping results of the calli. For example, in event (transgene‐positive calli) #4, seedlings regenerated include multiple phenotypes; in event #3, all seedlings regenerated were albino (Figure 3a,b and Table S1). In total, 66% of regenerated seedlings had a homozygous or bi‐allelic mutant phenotype (Table S1). The genotypes of the seedlings were also confirmed by RFLP assay of more than 10 seedlings selected from events #1–#4 (Figure S4). To further analyse the mutation types in the seedlings, the plants chosen for RFLP analysis were also selected for direct sequencing of the PCR products spanning the targeted site. The results revealed that all the albino seedlings were homozygous or bi‐allelic mutants, with most of the remainder being heterozygous or mosaic mutants (Table S2). Considering that the albino and chlorotic seedlings are homozygous or bi‐allelic mutants, the ratio of homozygous or bi‐allelic mutants in each event and total events was counted. For the target site in the *zb7* gene, the T0 plants regenerated were edited high efficiently with homozygous or bi‐allelic mutants accounting for about 66% (Table 1).

**Table 1:** Genotype statistics of transgenic T0 plants targeted by DPC CRISPR/Cas9 system at the *zb7* gene

Transgenic events	Homozygous & bi‐allelic mutants	Heterozygous & mosaic mutants	Total	Ratio of homozygous & bi‐allelic mutants
#1	29	10	39	74%
#2	1	19	20	5.0%
#3	41	0	41	100%
#4	18	3	21	86%
#5	9	0	9	100%
#6	16	2	18	89%
#7	19	0	19	100%
#8	1	13	14	7%
#9	16	12	28	57%
#10	6	22	28	21%
Total	156	81	237	66%

### DPC CRISPR/Cas9‐mediated targeted editing of another two sites in the T0 generation

**Figure 4:**
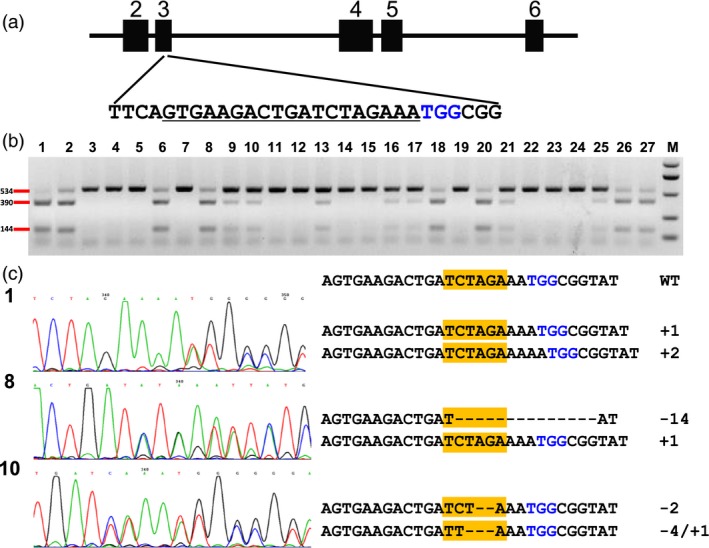
Identification of mutations in the *zyp1* gene by RFLP assay and Sanger sequencing. (a) Schematic illustration of the target site in the *zyp1* gene. Black box indicates exons, while the lines between them represent introns. Underlined sequence was selected for targeting; nucleotides marked in blue represent PAM. (b) RFLP assay of the genomic DNA of transgenic seedlings. Lane 1, control (wild‐type DNA amplicons digested with *Xba*I). Lane 2–27, 26 random selected transgenic seedlings. M, DNA marker. Primer pair zyp1‐F/zyp1‐R was used for PCR amplification, *Xba*I was used for digestion. (c) Sanger sequencing results of three seedlings. Left shows sequencing chromatograph, and right shows the edited sequences at the target site. Nucleotides marked in blue represent PAM. The yellow box indicates the *Xba*I site.

To test whether the DPC CRISPR/Cas9 system could generally mediate high‐efficiency genome editing in maize, we then chose another two target sites for targeting. One target site is located in the 3rd exon of the maize *zyp1* gene, which is reported to encode the central element protein of the synaptonemal complex (SC) and be functional in meiosis (Figure 4a; Barakate *et al*., 2014). The DPC CRISPR/Cas9 construct for targeting this site was transformed into maize immature embryos by Agrobacterium. In total, 26 seedlings, which were regenerated from three independent calli lines, were subjected to analysis. The RFLP assay indicated that 12 seedlings showed homozygous or bi‐allelic mutations (Figure 4b). We then directly subjected the PCR products for Sanger sequencing. The results showed that 23 seedlings were homozygous or bi‐allelic mutants (Table S3). The reason that the RFLP results differ with the Sanger sequencing is due to the mutations not disrupting the restriction site (Figure 4c). Most mutations are small insertions and deletions; mutations altering protein sequence but not causing a frameshift were identified. For this target site, the regenerated T0 plants were edited efficiently with 23 of 26 seedlings homozygous or bi‐allelic mutants.

**Figure 5:**
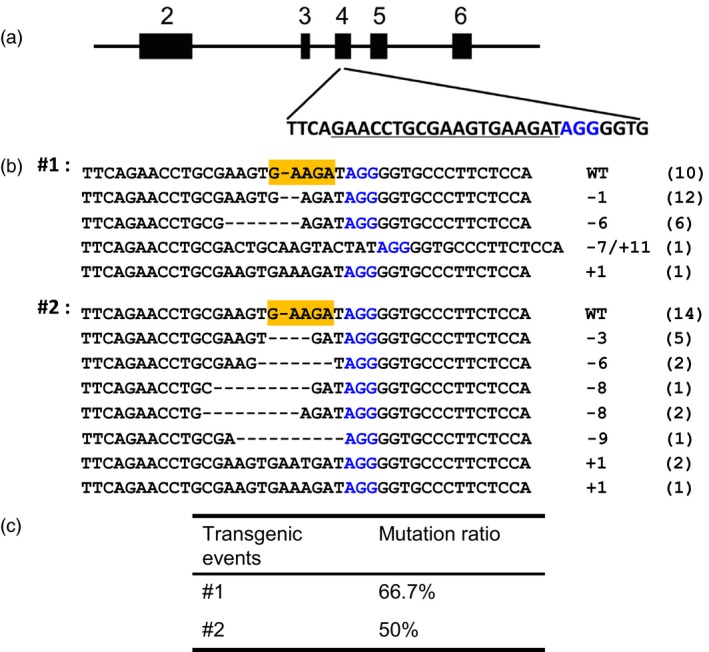
Mutation analysis of the *smc3* site targeted by DPC CRISPR/Cas9 system in transgenic calli. (a) Schematic illustration of the target site in the *smc3* gene. Black box indicates exons, while the line between them introns. Underlined sequence was selected for targeting; nucleotides marked in blue represent PAM. (b) Mutation analysis of the two transgene‐positive calli by cloning followed by Sanger sequencing. Sequence of the target site was amplified by PCR with primer pair smc3‐F/smc3‐R and cloned. Thirty clones were subjected to Sanger sequencing for each callus. The mutation types and number for different clones are shown on the right. (c) A table showing the ratio of mutant alleles in the two calli.

Another target site is located in the exonic region of the gene *smc3*, which has a putative role for chromosome structural maintenance and proved to be essential for embryo development (Figure 5a; Liu *et al*., 2002). The DPC CRISPR/Cas9 construct for targeting this gene was transformed into maize immature embryos by Agrobacterium. The transformation efficiency for the construct targeting this site is much lower (0.15%) compared with other constructs under the same conditions (1.5%). Only two transgene‐positive calli were obtained after a longer culture time (2 weeks) for selection due to the weak growth of the calli. The significantly lower transformation efficiency for this site suggested that knockout of this gene in maize calli may be lethal, implying that homozygous or bi‐allelic mutations occurred in most early transformed calli cells. To confirm that targeted mutation occurred in this site, we analysed the two transgene‐positive calli by genotyping. The results revealed that both the two calli are chimeras with multiple mutant alleles; the ratio of the mutant alleles in the two calli is 66.7% and 50%, respectively (Figure 5b,c).

Because a pretest of the activity of sgRNAs is helpful, we also tested whether the DPC CRISPR/Cas9 system can generate genome editing in protoplasts. The results showed that all the three targeted loci chosen could be edited. By Sanger sequencing of the site‐specific PCR amplicons, indels at the target loci were detected (Figure S5a,b).

### Transmission of the mutations to the next generation and generation of new mutations

**Figure 6:**
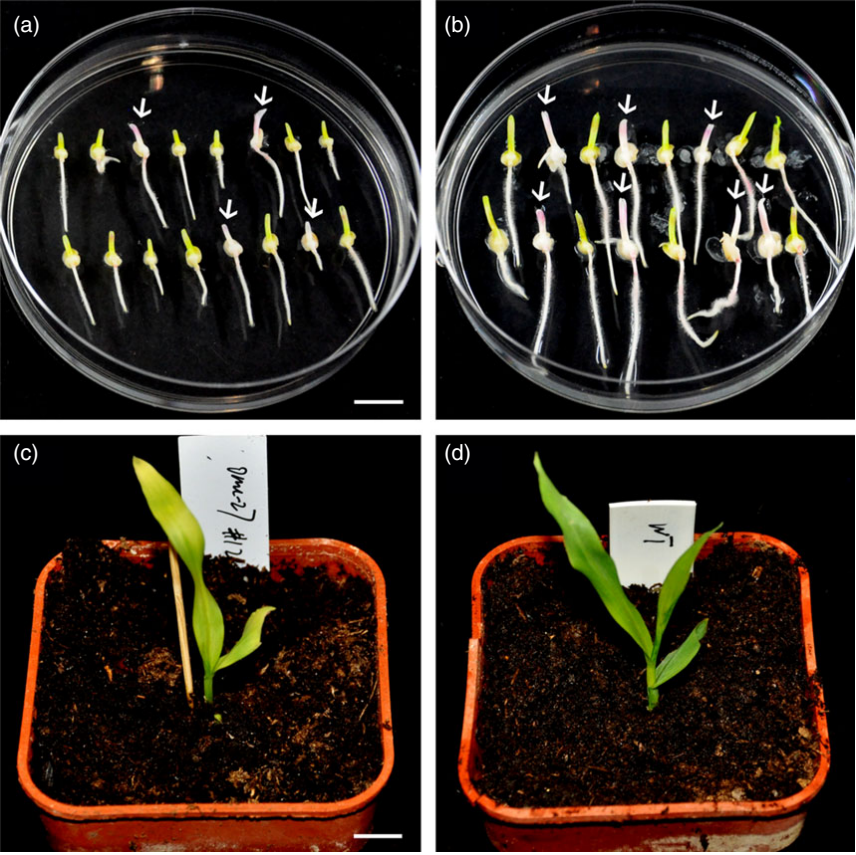
Phenotypes of the T1 seedlings by targeting the *zb7* gene using DPC CRISPR/Cas9 system. (a, b) Culture of immature embryos from two sibling ears of T0 transgenic plants. Arrows indicate albino seedlings. (c) One seedling shows a zebra phenotype which mimics a previously reported *zb7* mutant. (d) Control (regenerated wild‐type Hi‐II seedling). Bars = 1 cm.

As the homozygous or bi‐allelic mutants for the *zb7* gene can only survive a few weeks, the mutant alleles could not be transmitted. The mosaic seedlings with high mutation ratio also grow weakly and do not survive. To check whether the mutations created by the DPC CRISPR/Cas9 system could be transmitted to the offspring and new mutations could be generated, we planted the heterozygous seedlings and mosaic seedlings with low mutation ratios and performed crosses among them (Table S4). In five crosses among heterozygous plants and mosaic plants with low mutation ratios (<10%), immature embryos of the hybrid ears were cultured, and the genotype of T1 seedlings was analysed by RFLP assay and Sanger sequencing (Table S4). In crosses 1, 2 and 3, the mutant alleles of the T0 generation were transmitted to the T1 generation (Table S4). The results indicated that the mutations in T0 plants generated by the DPC CRISPR/Cas9 system could be stably inherited. In all five crosses, new alleles could be detected in T1 seedlings. The new alleles were also detected in Cas9‐free T1 seedlings (crosses 3 and 5), suggesting that mutations occurred in gametes of the T0 plant, which is consistent with our assumption. However, new alleles could also be generated in zygotes or early‐stage embryos. Most homozygous or bi‐allelic mutants recovered in the T1 generation showed an albino phenotype (Figure 6a,b). Interestingly, one plant shows a zebra phenotype, which mimics the phenotype of a mutant reported previously (Lu *et al*., 2012; Figure 6c,d). From examination in detail, we found that this *zb7* mutant protein has one tyrosine deletion combined with a substitution (glutamine to lysine; Figure S3b). In conclusion, the mutant alleles generated by the DPC CRISPR/Cas9 system could be stably transmitted to the next generation and furthermore, the gametes or early‐stage embryos in T0 plants could be edited.

### Off‐target analysis

**Figure 7:**
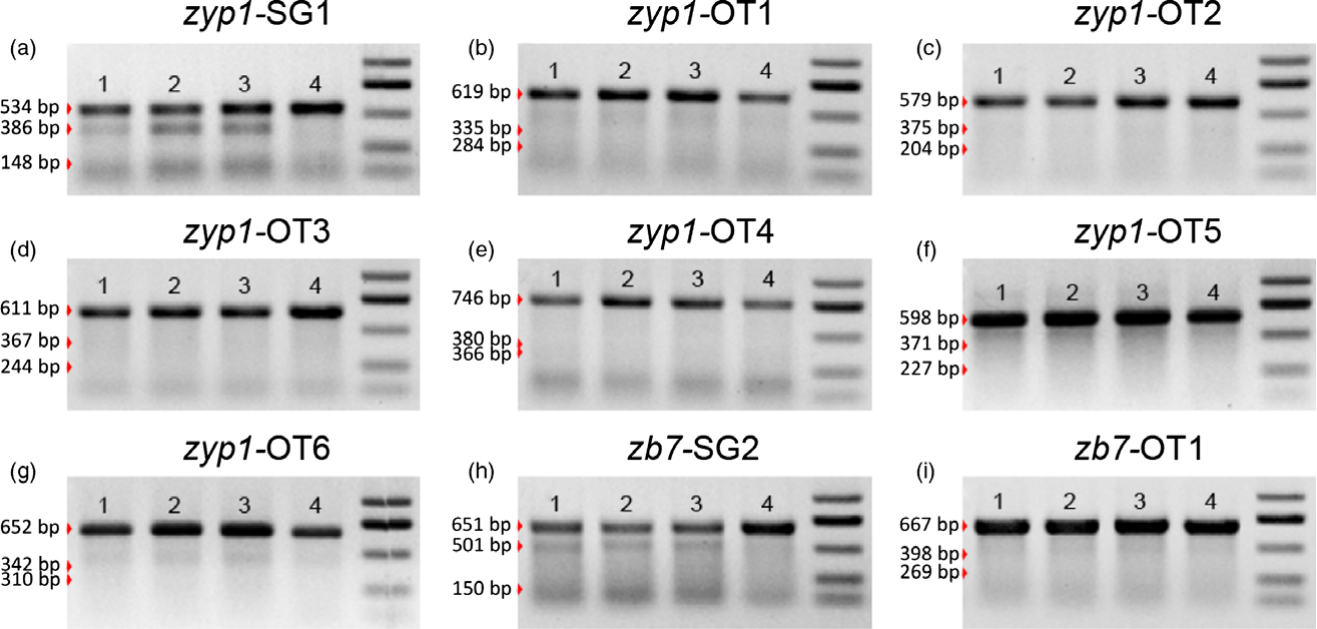
Off‐target analysis of the two loci by T7EI assay. (a) Target site in *zyp1* gene (*zyp1*‐SG1 in Table S8). (b–g) Predicted off‐target sites (*zyp1*‐OT1 to *zyp1*‐OT6 in Table S8). (h) Target site in *zb7* gene (*zb7‐* SG2 in Table S8). (i) Predicted off‐target sites (*zb7‐* OT1 in Table S8). For all sites, lanes 1–3 were three calli sample with homozygous or bi‐allelic mutation at the target sites and lane 4 is a control (wild‐type DNA sample used).

In addition to the editing efficiency, the specificity is also of concern when using CRISPR/Cas9 for targeted genome editing. In mammals, off‐target mutations were reported to take place in loci even with up to five nucleotide mismatch (Fu *et al*., 2013; Hsu *et al*., 2013; Pattanayak *et al*., 2013). To verify the specificity of using the DPC CRISPR/Cas9 system for targeted genome editing in maize, two *Cas9*‐positive T0 mutant plants with the *zb7* gene edited and one control T0 plant without *Cas9* were chosen for whole‐genome resequencing (Table S5). Approximately 11–14× average depth reads for each sample were sequenced with the Illumina Hiseq 2500 system (Table S5) and were examined for off‐target mutation. The resequencing data of *Cas9*‐positive and wild‐type plants were compared with the maize reference genome sequence. We first predicted the potential off‐target sites using online tools (Bae *et al*., 2014), over 1000 potential off‐target sites were identified with up to five mismatches. These potential off‐target sites were then checked with the resequencing data. The results show that no mutations were detected in these potential off‐target sites (Table S6). In contrast, the expected mutations in the target site of *zb7* were easily identified for the two *Cas9*‐positive T0 plants (Figure S6). The number of indels and SNPs was revealed genome‐wide. The slight difference of the number of indels and SNPs between the *Cas9*‐positive mutant samples and control sample was likely due to sequencing depth. (Table S7). The predicted off‐target sites for another two sites were also analysed by T7EI assay (Figure 7 and Table S8). No mutations could be detected for the potential off‐target sites.

## Discussion

To date, the CRISPR/Cas9 system has been widely adopted for genome editing in both monocots and dicots (Puchta, 2016). However, the efficiency of targeted genome editing is varied among species and different laboratories. Based on the reported results, the proper promoters used for both *Cas9* and sgRNA are important for genome editing (Ma *et al*., 2016). We report here the DPC CRISPR/Cas9 system is highly efficient for targeted genome editing in maize. In this system, *Cas9* and sgRNA are driven by the maize *dmc1* promoter and *U3* promoter, respectively. The mutation efficiency in transgene‐positive calli is 100%. Our results uncovered a new maize promoter that could be used for highly efficient genome editing in maize, thus providing further evidence that the efficiency of genome editing could be enhanced by screening and testing of promoters for *Cas9* and sgRNA.

Previously the *dmc1* gene was thought to be specifically expressed in reproductive tissue, and the *dmc1* promoter would be meiosis specific. This gene is widely conserved in eukaryotes (Kagawa and Kurumizaka, 2010). By expression analysis, our results revealed that the *dmc1* promoter could drive Cas9 to be highly expressed in callus, at least in maize. This was also confirmed by expression analysis of the endogenous *dmc1* gene in different maize tissues including callus. The *dmc1* gene has the highest expression level in tassel, but is also highly expressed in callus. These data could probably throw light on how the DPC CRISRP/Cas9 system can mediate targeted genome editing so efficiently.

It had been reported that the mutation efficiency of the vectors using *ubiquitin* promoter‐driven *Cas9* combined with *U6*‐driven sgRNAs was more than 77% for three target sites when the constructs were delivered using biolistic‐mediated transformation (Svitashev *et al*., 2015). The biolistic transformation usually led to high copy number of transgenes integrated into the genomic region, not to mention the higher initial copy numbers of the plasmids delivered into the recipient cells. The high mutation efficiency may more or less benefit from the high expression level of the *Cas9* gene caused by the high copy number of the transgene. The very similar constructs used by another group only generated a low mutation efficiency when using Agrobacterium for plasmid delivery (Zhu *et al*., 2016). Recently another group reported high‐efficiency targeted genome editing in maize Hi‐II germplasm using Agrobacterium‐mediated transformation (Char *et al*., 2016). The *ubiquitin* promoter is also used for driving *Cas9* expression, and two sgRNAs driven by rice *U6* promoters together were used for each gene to be targeted. The bi‐allelic mutant ratio is 21.3%–57.4% for the four genes targeted. Our results show that using the *dmc1* promoter‐driven *Cas9* combined with one sgRNA, the transgene‐positive calli were edited at the target loci at an efficiency of 100% with bi‐allelic mutations present in up to 66% of the regenerated seedlings. Thus, with a pretest of the activity of the sgRNAs in protoplasts, homozygous mutants for a specific gene could be obtained in the T0 generation at high efficiency using the DPC CRISPR/Cas9 system with one sgRNA. High‐efficiency multiplex genome editing in T0 generation is expected.

In most reports, sites in the gene coding region were chosen for applying the CRISPR/Cas9 to perform targeted genome editing, and usually the outcome is gene knockout. Recently, one group reported the results of editing the promoter region of a gene and generated quantitative trait variation with improved traits in tomato (Rodriguez‐Leal *et al*., 2017). By randomly selecting a target site in the *zb7* gene, we obtained two kinds of mutant alleles with the coding sequence altered that show interesting phenotypes. Our results provide evidence that editing of the gene coding region without causing frameshift is probably also be useful for improving crop traits. It is also suggested that functional genomics could be explored deeper by altering the coding sequence of a gene rather than just making a knockout.

Except for the on‐target efficiency for CRISPR/Cas9‐mediated genome editing, another concern is the off‐target frequency. In mammals, the off‐target mutation frequency of the CRISPR/Cas9 system has been proven to be high when similar sites exist in the genome, especially sites with the same PAM‐proximal seed sequences. Off‐target mutation occurred in loci with even five mismatches. We conducted whole‐genome resequencing to detect off‐target mutation in the edited T0 plants. The results indicated no off‐target mutations occurred in the predicted sites with three or more mismatches.

In our experiments, using the *dmc1* promoter‐controlled *Cas9* system, high‐efficiency genome editing at the target site was identified in maize. The editing efficiency could be further improved by using more sgRNAs for one target site or by optimizing the criteria for sgRNA design (Dang *et al*., 2015; Doench *et al*., 2016; Wong *et al*., 2015). It is worth mentioning that previous studies have indicated that a maize codon‐optimized Cas9 could boost the mutation efficiency compared with a human codon‐optimized Cas9 (Xing *et al*., 2014). As the Cas9 used in our DPC CRISPR/Cas9 system is human codon‐optimized, we suggest that the targeted genome editing efficiency could be further increased using a plant or maize codon‐optimized Cas9. The higher genome editing efficiency could also largely benefit genome‐wide targeted mutagenesis in maize. Lastly, although at present we tested this system in maize, due to the evolutionary conservation of the *dmc1* gene, we suggest that the *dmc1* promoter‐controlled CRISPR/Cas9 system may be effective for other plant species.

## Methods

### Constructs and bacterial strains

The maize *dmc1* promoter was amplified using primer pair dmcp‐F and dmcp‐R (Table S10) with B73 genomic DNA as template. The plasmid *35S*‐*Cas9*‐SK (Feng *et al*., 2013) was digested with the restriction enzyme *Xho*I (NEB) to release the *35S* promoter. Both the products were run in an agarose gel and then purified using a DNA purification Kit (TianGen). The purified linear fragments were assembled using the ‘Easygeno Assembly Cloning Kit’ (TianGen) to generate *Dmc1*‐*Cas9*‐SK. This plasmid was further digested by *Xma*I and *Eco*RI to release the *Dmc1*‐*Cas9*‐Nos cassette; the released cassette was then inserted into the binary vector pTF101.1 to make p*Dmc1*‐Cas9 (Figure S7). The construction of the sgRNA cassette was described previously (Feng *et al*., 2016). The sgRNA cassette was amplified using primer pair sgRNA‐F and sgRNA‐R (Table S10), and then assembled into p*Dmc1*‐Cas9 with the ‘Easygeno Assembly Cloning Kit’ (TianGen). The bacterial strains used in this study include DH5α (*Escherichia coli*) for conventional cloning and EHA105 (Agrobacterium) for maize transformation.

### Agrobacterium‐mediated maize transformation

Maize Hi‐II germplasm was used as the transformation receptor. The seeds were planted in the experimental field in Beijing from May to September. The immature embryos were used for Agrobacterium‐mediated transformation following previous protocols (Frame *et al*., 2002). Tissue culture was performed in growth chambers in the dark. The regenerated plants were grown in chambers with a 16‐h light/8‐h dark condition at 25°C.

### DNA extraction, PCR verification and genotyping

Genomic DNA of the maize calli, seedlings and protoplasts was extracted with Plant Genomic DNA Kit (TianGen Biotech., Beijing, China). The transgenic analysis was performed by PCR with specific primer pair Cas9‐F/Cas9‐R (Table S10). The PCR amplicons spanning the target sites were either subjected to direct Sanger sequencing, or cloned into the pMD19‐T vector (Takara Biomedical Technology, Beijing, China) and followed by Sanger sequencing. The chromatogram files were analysed by online tools (Liu *et al*., 2015).

### RFLP assay and T7EI assay

Genome DNA that covered each target site was amplified from independent samples. Primers used were listed in Table S10. For RFLP assay, about 500 ng purified PCR products of each sample was digested with the corresponding enzyme (NEB, Ipswich, MA), the reaction products were analysed with 1.0% agarose gel electrophoresis. For the T7EI assay, PCR products obtained from the transgenic samples were mixed with the respective product derived from wild type, denatured (95°C for 5 min) and reannealed (ramp down from 95°C to 80°C at 5°C/min, from 80°C to 25°C at 5°C/2 min, hold on at 4°C). The reannealed products were then used to detect mutation with T7 endonuclease I (NEB, Ipswich, MA). The reaction products were analysed with 1.0% agarose gel electrophoresis.

### Maize protoplast transformation

Maize B73 inbred seeds were germinated in the dark at 30°C for 3 days and then moved to a dark chamber at 25°C for a week. The fresh leaves of the seedlings were used to isolate mesophyll protoplasts. Maize protoplast transformation was carried out according to previously reported methods (Feng *et al*., 2016).

### Whole‐genome resequencing and data analysis

Total DNA was isolated from the leaves of two *Cas9*‐positive transgenic plants and one wild‐type plant for genome resequencing. The genome sequence libraries were constructed according to the standard Illumina protocols and were applied to HiSeq 2500 system (Illumina, San Diego, CA). The whole‐genome resequencing data sets generated in this study are available from the NCBI BioProject (https://www.ncbi.nlm.nih.gov/bioproject) under accession number PRJNA416839. The SNPs and indels were identified using the GATK Best Practices (Van der Auwera *et al*., 2013). The potential off‐target sites were identified with Cas‐OFFinder web tool with default parameters (Bae *et al*., 2014).

## Conflict of interest

The authors declare competing financial interests: the authors have filed a patent application based on the results reported in this article.

## Supporting information


**Figure S1** Comparison of the mutation efficiency between the two maize polIII promoters.
**Figure S2** Expression analysis of the *Cas9* and *dmc1* gene.
**Figure S3** Detailed mutation analysis of the plants with chlorotic and zebra phenotypes.
**Figure S4** Genotyping of the transgenic T0 plants by RFLP assay.
**Figure S5** Mutation analysis of the three sites targeted by DPC CRISPR/Cas9 system in protoplasts.
**Figure S6** Integrative Genomics Viewer (IGV) snapshots of the target site in the *zb7* gene.
**Figure S7** DNA sequence of the p*Dmc1*‐Cas9 binary vector.

**Table S1** Phenotype statistics of transgenic T0 plants targeted by DPC CRISPR/Cas9 system.
**Table S2** Genotyping of the T0 plants by direct‐sequencing of the PCR products.
**Table S3** Genotype statistics of transgenic T0 plants targeted by DPC CRISPR/Cas9 system at *zyp1* gene.
**Table S4** Genotyping of the T1 plants by RFLP assay and sanger sequencing at the *zb7* gene.
**Table S5** Summary of the re‐sequencing quality of different maize transgenic lines.
**Table S6** Summary of the putative off‐target examination.
**Table S7** Summary of the mutations of the whole genome re‐sequencing of maize transgenic lines and control.
**Table S8** Predicted CRISPR/Cas9 off‐target sites of the two gene *zyp1* and *zb7*.
**Table S9** Maize genes targeted in this study.
**Table S10** Primers used in this study.

## References

[pbi12920-bib-0001] Bae, S. , Park, J. and Kim, J.‐S. (2014) Cas‐OFFinder: a fast and versatile algorithm that searches for potential off‐target sites of Cas9 RNA‐guided endonucleases. Bioinformatics, 30, 1473–1475. 24463181 10.1093/bioinformatics/btu048 PMC4016707

[pbi12920-bib-0002] Barakate, A. , Higgins, J.D. , Vivera, S. , Stephens, J. , Perry, R.M. , Ramsay, L. , Colas, I. *et al* (2014) The synaptonemal complex protein ZYP1 is required for imposition of meiotic crossovers in barley. Plant Cell, 26, 729–740. 24563202 10.1105/tpc.113.121269 PMC3967036

[pbi12920-bib-0003] Brooks, C. , Nekrasov, V. , Lippman, Z.B. and Van Eck, J. (2014) Efficient gene editing in tomato in the first generation using the clustered regularly interspaced short palindromic repeats/CRISPR‐associated 9 system. Plant Physiol. 166, 1292–1297. 25225186 10.1104/pp.114.247577 PMC4226363

[pbi12920-bib-0004] Char, S.N. , Neelakandan, A.K. , Nahampun, H. , Frame, B. , Main, M. , Spalding, M.H. , Becraft, P.W. *et al* (2016) An Agrobacterium‐delivered CRISPR/Cas9 system for high‐frequency targeted mutagenesis in maize. Plant Biotechnol. J. 15, 257–268. 27510362 10.1111/pbi.12611 PMC5259581

[pbi12920-bib-0005] Cong, L. , Ran, F.A. , Cox, D. , Lin, S. , Barretto, R. , Habib, N. , Hsu, P.D. *et al* (2013) Multiplex genome engineering using CRISPR/Cas systems. Science, 339, 819–823. 23287718 10.1126/science.1231143 PMC3795411

[pbi12920-bib-0006] Dang, Y. , Jia, G. , Choi, J. , Ma, H. , Anaya, E. , Ye, C. , Shankar, P. *et al* (2015) Optimizing sgRNA structure to improve CRISPR‐Cas9 knockout efficiency. Genome Biol. 16, 280. 26671237 10.1186/s13059-015-0846-3 PMC4699467

[pbi12920-bib-0007] DiCarlo, J.E. , Norville, J.E. , Mali, P. , Rios, X. , Aach, J. and Church, G.M. (2013) Genome engineering in *Saccharomyces cerevisiae* using CRISPR‐Cas systems. Nucleic Acids Res. 41, 4336–4343. 23460208 10.1093/nar/gkt135 PMC3627607

[pbi12920-bib-0008] Doench, J.G. , Fusi, N. , Sullender, M. , Hegde, M. , Vaimberg, E.W. , Donovan, K.F. , Smith, I. *et al* (2016) Optimized sgRNA design to maximize activity and minimize off‐target effects of CRISPR‐Cas9. Nat. Biotechnol. 34, 184–191. 26780180 10.1038/nbt.3437 PMC4744125

[pbi12920-bib-0009] Feng, Z. , Zhang, B. , Ding, W. , Liu, X. , Yang, D.L. , Wei, P. , Cao, F. *et al* (2013) Efficient genome editing in plants using a CRISPR/Cas system. Cell Res. 23, 1229–1232. 23958582 10.1038/cr.2013.114 PMC3790235

[pbi12920-bib-0010] Feng, Z. , Mao, Y. , Xu, N. , Zhang, B. , Wei, P. , Yang, D.L. , Wang, Z. *et al* (2014) Multigeneration analysis reveals the inheritance, specificity, and patterns of CRISPR/Cas‐induced gene modifications in *Arabidopsis* . Proc. Natl Acad. Sci. USA, 111, 4632–4637. 24550464 10.1073/pnas.1400822111 PMC3970504

[pbi12920-bib-0011] Feng, C. , Yuan, J. , Wang, R. , Liu, Y. , Birchler, J.A. and Han, F. (2016) Efficient targeted genome modification in maize using CRISPR/Cas9 system. J. Genet. Genomics, 43, 37–43. 26842992 10.1016/j.jgg.2015.10.002

[pbi12920-bib-0500] Frame, B.R. , Shou, H. , Chikwamba, R.K. , Zhang, Z. , Xiang, C. , Fonger, T.M. , Pegg, S.E.K. , *et al* (2002) Agrobacterium tumefaciens‐mediated transformation of maize embryos using a standard binary vector system. Plant Physiol. 129, 13–22. 12011333 10.1104/pp.000653 PMC1540222

[pbi12920-bib-0012] Friedland, A.E. , Tzur, Y.B. , Esvelt, K.M. , Colaiacovo, M.P. , Church, G.M. and Calarco, J.A. (2013) Heritable genome editing in *C. elegans* via a CRISPR‐Cas9 system. Nat. Methods, 10, 741–743. 23817069 10.1038/nmeth.2532 PMC3822328

[pbi12920-bib-0013] Fu, Y. , Foden, J.A. , Khayter, C. , Maeder, M.L. , Reyon, D. , Joung, J.K. and Sander, J.D. (2013) High‐frequency off‐target mutagenesis induced by CRISPR‐Cas nucleases in human cells. Nat. Biotechnol. 31, 822–826. 23792628 10.1038/nbt.2623 PMC3773023

[pbi12920-bib-0014] Gaj, T. , Gersbach, C.A. and Barbas, C.F. III . (2013) ZFN, TALEN, and CRISPR/Cas‐based methods for genome engineering. Trends Biotechnol. 31, 397–405. 23664777 10.1016/j.tibtech.2013.04.004 PMC3694601

[pbi12920-bib-0015] Gratz, S.J. , Cummings, A.M. , Nguyen, J.N. , Hamm, D.C. , Donohue, L.K. , Harrison, M.M. , Wildonger, J. *et al* (2013) Genome Engineering of *Drosophila* with the CRISPR RNA‐Guided Cas9 Nuclease. Genetics, 194, 1029–1035. 23709638 10.1534/genetics.113.152710 PMC3730909

[pbi12920-bib-0016] Hsu, P.D. , Scott, D.A. , Weinstein, J.A. , Ran, F.A. , Konermann, S. , Agarwala, V. , Li, Y. *et al* (2013) DNA targeting specificity of RNA‐guided Cas9 nucleases. Nat. Biotechnol. 31, 827–832. 23873081 10.1038/nbt.2647 PMC3969858

[pbi12920-bib-0017] Hsu, P.D. , Lander, E.S. and Zhang, F. (2014) Development and applications of CRISPR‐Cas9 for genome engineering. Cell, 157, 1262–1278. 24906146 10.1016/j.cell.2014.05.010 PMC4343198

[pbi12920-bib-0018] Hwang, W.Y. , Fu, Y.F. , Reyon, D. , Maeder, M.L. , Tsai, S.Q. , Sander, J.D. , Peterson, R.T. *et al* (2013) Efficient genome editing in zebrafish using a CRISPR‐Cas system. Nat. Biotechnol. 31, 227–229. 23360964 10.1038/nbt.2501 PMC3686313

[pbi12920-bib-0019] Jiang, W.Y. , Bikard, D. , Cox, D. , Zhang, F. and Marraffini, L.A. (2013) RNA‐guided editing of bacterial genomes using CRISPR‐Cas systems. Nat. Biotechnol. 31, 233–239. 23360965 10.1038/nbt.2508 PMC3748948

[pbi12920-bib-0020] Kagawa, W. and Kurumizaka, H. (2010) From meiosis to postmeiotic events: uncovering the molecular roles of the meiosis‐specific recombinase Dmc1. FEBS J. 277, 590–598. 20015079 10.1111/j.1742-4658.2009.07503.x

[pbi12920-bib-0021] Klimyuk, V.I. and Jones, J.D. (1997) AtDMC1, the *Arabidopsis* homologue of the yeast DMC1 gene: characterization, transposon‐induced allelic variation and meiosis‐associated expression. Plant J. 11, 1–14. 9025299 10.1046/j.1365-313x.1997.11010001.x

[pbi12920-bib-0022] Leader, D.J. , Connelly, S. , Filipowicz, W. and Brown, J.W. (1994) Characterisation and expression of a maize U3 snRNA gene. Biochim. Biophys. Acta, 1219, 145–147. 7522055 10.1016/0167-4781(94)90257-7

[pbi12920-bib-0023] Li, D.L. , Qiu, Z.W. , Shao, Y.J. , Chen, Y.T. , Guan, Y.T. , Liu, M. , Li, Y. *et al* (2013a) Heritable gene targeting in the mouse and rat using a CRISPR‐Cas system. Nat. Biotechnol. 31, 681–683. 23929336 10.1038/nbt.2661

[pbi12920-bib-0024] Li, J.F. , Norville, J.E. , Aach, J. , McCormack, M. , Zhang, D. , Bush, J. , Church, G.M. *et al* (2013b) Multiplex and homologous recombination‐mediated genome editing in *Arabidopsis* and *Nicotiana benthamiana* using guide RNA and Cas9. Nat. Biotechnol. 31, 688–691. 23929339 10.1038/nbt.2654 PMC4078740

[pbi12920-bib-0025] Li, W. , Teng, F. , Li, T.D. and Zhou, Q. (2013c) Simultaneous generation and germline transmission of multiple gene mutations in rat using CRISPR‐Cas systems. Nat. Biotechnol. 31, 684–686. 23929337 10.1038/nbt.2652

[pbi12920-bib-0026] Li, C. , Liu, C. , Qi, X. , Wu, Y. , Fei, X. , Mao, L. , Chen, B. *et al* (2017) RNA‐guided Cas9 as an in vivo desired‐target mutator in maize. Plant Biotechnol. J. 15, 1566–1576. 28379609 10.1111/pbi.12739 PMC5698053

[pbi12920-bib-0027] Liang, Z. , Zhang, K. , Chen, K. and Gao, C. (2014) Targeted mutagenesis in Zea mays using TALENs and the CRISPR/Cas system. J. Genet. Genomics, 41, 63–68. 24576457 10.1016/j.jgg.2013.12.001

[pbi12920-bib-0028] Liu, C.M. , McElver, J. , Tzafrir, I. , Joosen, R. , Wittich, P. , Patton, D. , Van Lammeren, A.A. *et al* (2002) Condensin and cohesin knockouts in *Arabidopsis* exhibit a titan seed phenotype. Plant J. 29, 405–415. 11846874 10.1046/j.1365-313x.2002.01224.x

[pbi12920-bib-0029] Liu, W. , Xie, X. , Ma, X. , Li, J. , Chen, J. and Liu, Y.G. (2015) DSDecode: a web‐based tool for decoding of sequencing chromatograms for genotyping of targeted mutations. Mol. Plant, 8, 1431–1433. 26032088 10.1016/j.molp.2015.05.009

[pbi12920-bib-0030] Lu, X.M. , Hu, X.J. , Zhao, Y.Z. , Song, W.B. , Zhang, M. , Chen, Z.L. , Chen, W. *et al* (2012) Map‐based cloning of zb7 encoding an IPP and DMAPP synthase in the MEP pathway of maize. Mol. Plant, 5, 1100–1112. 22498772 10.1093/mp/sss038

[pbi12920-bib-0031] Ma, X. , Zhang, Q. , Zhu, Q. , Liu, W. , Chen, Y. , Qiu, R. , Wang, B. *et al* (2015) A robust CRISPR/Cas9 system for convenient, high‐efficiency multiplex genome editing in monocot and dicot plants. Mol. Plant, 8, 1274–1284. 25917172 10.1016/j.molp.2015.04.007

[pbi12920-bib-0032] Ma, X. , Zhu, Q. , Chen, Y. and Liu, Y.G. (2016) CRISPR/Cas9 platforms for genome editing in plants: developments and applications. Mol. Plant, 9, 961–974. 27108381 10.1016/j.molp.2016.04.009

[pbi12920-bib-0033] Mali, P. , Yang, L. , Esvelt, K.M. , Aach, J. , Guell, M. , DiCarlo, J.E. , Norville, J.E. *et al* (2013) RNA‐guided human genome engineering via Cas9. Science, 339, 823–826. 23287722 10.1126/science.1232033 PMC3712628

[pbi12920-bib-0034] Miao, J. , Guo, D. , Zhang, J. , Huang, Q. , Qin, G. , Zhang, X. , Wan, J. *et al* (2013) Targeted mutagenesis in rice using CRISPR‐Cas system. Cell Res. 23, 1233–1236. 23999856 10.1038/cr.2013.123 PMC3790239

[pbi12920-bib-0035] Nannas, N.J. and Dawe, R.K. (2015) Genetic and genomic toolbox of *Zea mays* . Genetics, 199, 655–669. 25740912 10.1534/genetics.114.165183 PMC4349061

[pbi12920-bib-0036] Nekrasov, V. , Staskawicz, B. , Weigel, D. , Jones, J.D. and Kamoun, S. (2013) Targeted mutagenesis in the model plant *Nicotiana benthamiana* using Cas9 RNA‐guided endonuclease. Nat. Biotechnol. 31, 691–693. 23929340 10.1038/nbt.2655

[pbi12920-bib-0037] Pattanayak, V. , Lin, S. , Guilinger, J.P. , Ma, E. , Doudna, J.A. and Liu, D.R. (2013) High‐throughput profiling of off‐target DNA cleavage reveals RNA‐programmed Cas9 nuclease specificity. Nat. Biotechnol. 31, 839–843. 23934178 10.1038/nbt.2673 PMC3782611

[pbi12920-bib-0038] Puchta, H. (2016) Applying CRISPR/Cas for genome engineering in plants: the best is yet to come. Curr. Opin. Plant Biol. 36, 1–8. 27914284 10.1016/j.pbi.2016.11.011

[pbi12920-bib-0039] Puchta, H. and Fauser, F. (2014) Synthetic nucleases for genome engineering in plants: prospects for a bright future. Plant J. 78, 727–741. 24112784 10.1111/tpj.12338

[pbi12920-bib-0040] Rodriguez‐Leal, D. , Lemmon, Z.H. , Man, J. , Bartlett, M.E. and Lippman, Z.B. (2017) Engineering quantitative trait variation for crop improvement by genome editing. Cell 171, 470–480. 28919077 10.1016/j.cell.2017.08.030

[pbi12920-bib-0041] Shan, Q.W. , Wang, Y.P. , Li, J. , Zhang, Y. , Chen, K.L. , Liang, Z. , Zhang, K. *et al* (2013) Targeted genome modification of crop plants using a CRISPR‐Cas system. Nat. Biotechnol. 31, 686–688. 23929338 10.1038/nbt.2650

[pbi12920-bib-0042] Svitashev, S. , Young, J.K. , Schwartz, C. , Gao, H. , Falco, S.C. and Cigan, A.M. (2015) Targeted mutagenesis, precise gene editing, and site‐specific gene insertion in maize using Cas9 and guide RNA. Plant Physiol. 169, 931–945. 26269544 10.1104/pp.15.00793 PMC4587463

[pbi12920-bib-0043] Van der Auwera, G.A. , Carneiro, M.O. , Hartl, C. , Poplin, R. , Del Angel, G. , Levy‐Moonshine, A. , Jordan, T. *et al* (2013) From FastQ data to high confidence variant calls: the Genome Analysis Toolkit best practices pipeline. Curr. Protoc. Bioinformatics, 43, 11.10.1‐33. 25431634 10.1002/0471250953.bi1110s43 PMC4243306

[pbi12920-bib-0044] Wang, Z.P. , Xing, H.L. , Dong, L. , Zhang, H.Y. , Han, C.Y. , Wang, X.C. and Chen, Q.J. (2015) Egg cell‐specific promoter‐controlled CRISPR/Cas9 efficiently generates homozygous mutants for multiple target genes in *Arabidopsis* in a single generation. Genome Biol. 16, 144. 26193878 10.1186/s13059-015-0715-0 PMC4507317

[pbi12920-bib-0045] Wong, N. , Liu, W. and Wang, X. (2015) WU‐CRISPR: characteristics of functional guide RNAs for the CRISPR/Cas9 system. Genome Biol. 16, 218. 26521937 10.1186/s13059-015-0784-0 PMC4629399

[pbi12920-bib-0046] Xie, K. and Yang, Y. (2013) RNA‐guided genome editing in plants using a CRISPR‐Cas system. Mol. Plant, 6, 1975–1983. 23956122 10.1093/mp/sst119

[pbi12920-bib-0047] Xie, K. , Minkenberg, B. and Yang, Y. (2015) Boosting CRISPR/Cas9 multiplex editing capability with the endogenous tRNA‐processing system. Proc. Natl Acad. Sci. USA, 112, 3570–3575. 25733849 10.1073/pnas.1420294112 PMC4371917

[pbi12920-bib-0048] Xing, H.L. , Dong, L. , Wang, Z.P. , Zhang, H.Y. , Han, C.Y. , Liu, B. , Wang, X.C. *et al* (2014) A CRISPR/Cas9 toolkit for multiplex genome editing in plants. BMC Plant Biol. 14, 327. 25432517 10.1186/s12870-014-0327-y PMC4262988

[pbi12920-bib-0049] Xu, R. , Li, H. , Qin, R. , Wang, L. , Li, L. , Wei, P. and Yang, J. *et al* (2014) Gene targeting using the *Agrobacterium tumefaciens*‐mediated CRISPR‐Cas system in rice. Rice, 7, 5. 24920971 10.1186/s12284-014-0005-6 PMC4052633

[pbi12920-bib-0050] Yu, Z.S. , Ren, M.D. , Wang, Z.X. , Zhang, B. , Rong, Y.K.S. , Jiao, R. and Gao, G. (2013) Highly efficient genome modifications mediated by CRISPR/Cas9 in *Drosophila* . Genetics, 195, 289–291. 23833182 10.1534/genetics.113.153825 PMC3761309

[pbi12920-bib-0051] Zhang, H. , Zhang, J. , Wei, P. , Zhang, B. , Gou, F. , Feng, Z. , Mao, Y. *et al* (2014) The CRISPR/Cas9 system produces specific and homozygous targeted gene editing in rice in one generation. Plant Biotechnol. J. 12, 797–807. 24854982 10.1111/pbi.12200

[pbi12920-bib-0052] Zhu, J. , Song, N. , Sun, S. , Yang, W. , Zhao, H. , Song, W. and Lai, J. (2016) Efficiency and inheritance of targeted mutagenesis in maize using CRISPR‐Cas9. Plant Physiol. 43, 25–36. 10.1016/j.jgg.2015.10.006 26842991

