# Supplementary material for: High‐efficiency genome editing using a dmc1 promoter‐controlled CRISPR/Cas9 system in maize
Source: Plant Biotechnol J. 2018 Apr 30;16(11):1848–57. doi: 10.1111/pbi.12920 (PMC6181213; doi:10.1111/pbi.12920)
Supplement: Supplementary file 1 — Figure S1 Comparison of the mutation efficiency between the two maize polIII promoters. Figure S2 Expression analysis of the Cas9 and dmc1 gene. Figure S3 Detailed mutation analysis of the plants with chlorotic and zebra phenotypes. Figure S4 Genotyping of the transgenic T0 plants by RFLP assay. Figure S5 Mutation analysis of the three sites targeted by DPC CRISPR/Cas9 system in protoplasts. Figure S6 Integrative Genomics Viewer (IGV) snapshots of the target site in the zb7 gene. Figure S7 DNA sequence of the pDmc1‐Cas9 binary vector. [file PBI-16-1848-s002.docx]

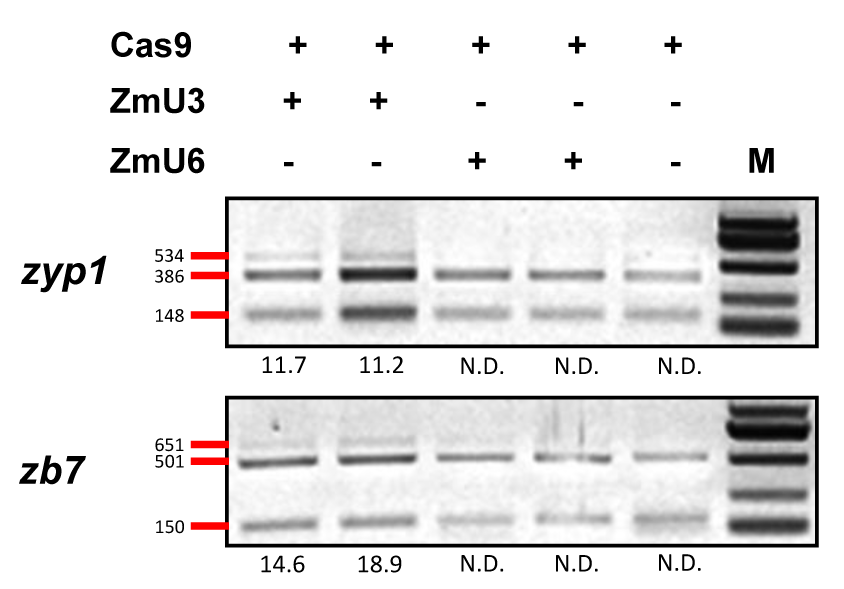


**Figure S1 Comparison of the mutation efficiency between the two maize polIII promoters.**

Two target sites located in the *zb7* and *zyp1* genes (see figure 2 and figure 4) were used for study. The sgRNAs driven by *U3* or *U6* were co-transformed with plasmids expressing Cas9. Two duplications were made for each transformation. M, DNA marker. N.D., not determined.


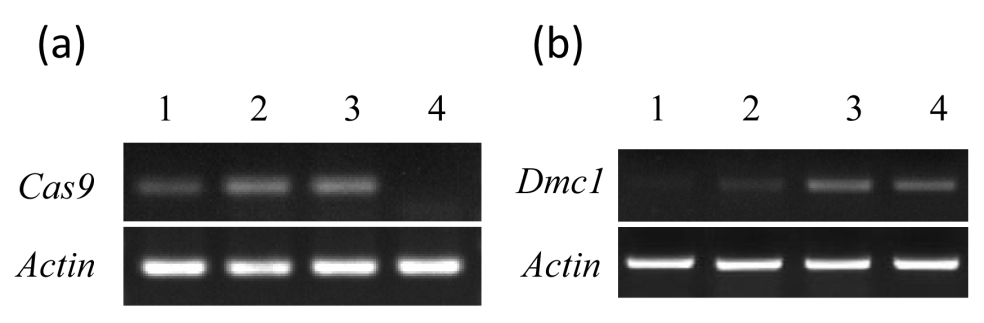


**Figure S2 Expression analysis of the *Cas9* and *dmc1* gene.**

(a) Analysis of Cas9 expression in three calli (#1-#3, *zb7* targeting) by RT-PCR using primer pair Cas9-F/Cas9-R. Lane 1-3, three calli; lane 4, control (callus sample without *Cas9* transgene). (b) Analysis of maize *dmc1* expression in calli and other tissues by RT-PCR with primer pair dmc1-F/dmc1-R. Lane 1-4, leaf, root, tassel, and callus. *Actin* was used as control gene.


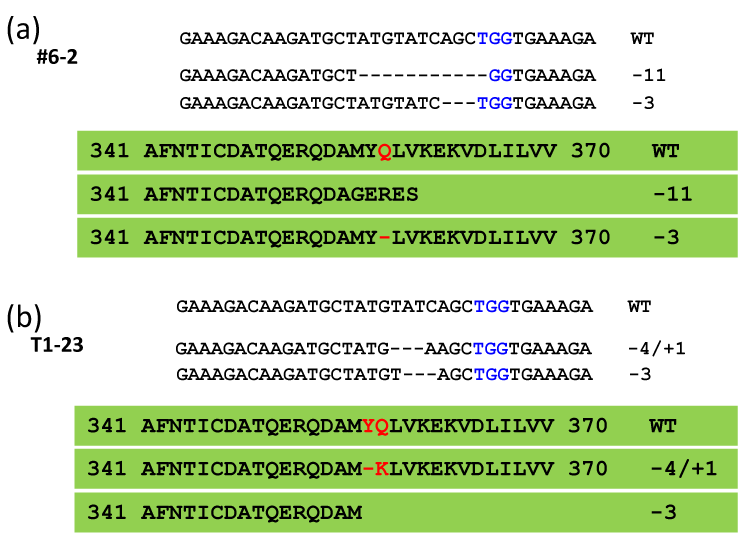


**Figure S3 Detailed mutation analysis of the plants with chlorotic and zebra phenotypes.**

(a) Sequence analysis of one T0 seedling with chlorotic phenotype. (b) Sequence analysis of one T1 seedling with zebra phenotype. Nucleotides marked in blue represent PAM. Sequences in green box are amino acid sequences at the target site region. For both part (a) and (b), the three amino acids sequences are corresponding to the DNA sequences listed.


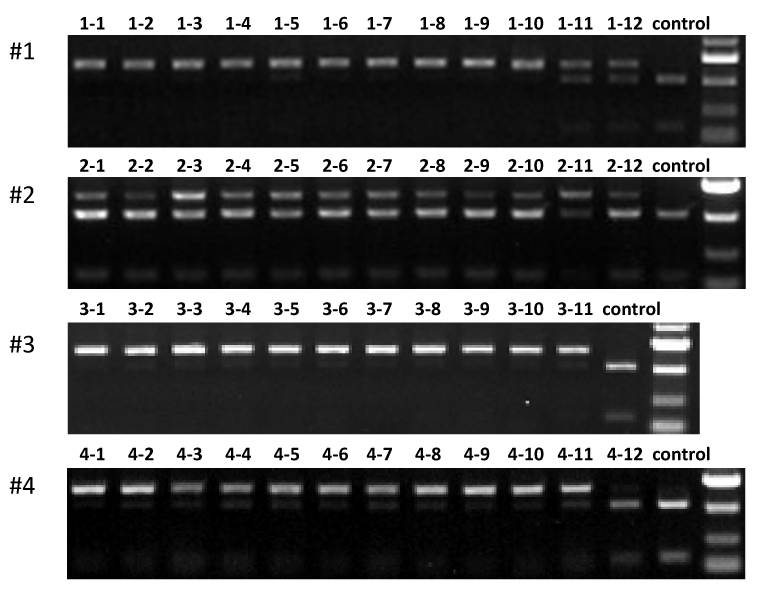


**Figure S4 Genotyping of the transgenic T0 plants by RFLP assay.**

RFLP assay of random selected seedlings regenerated from the four transgene-positive calli (#1-#4) in the first round of transformation. For each callus event, over 10 seedlings were analyzed. The primers and restriction enzyme used is the same as shown in Figure 2. Control, regenerated seedling sample without Cas9 transgene.


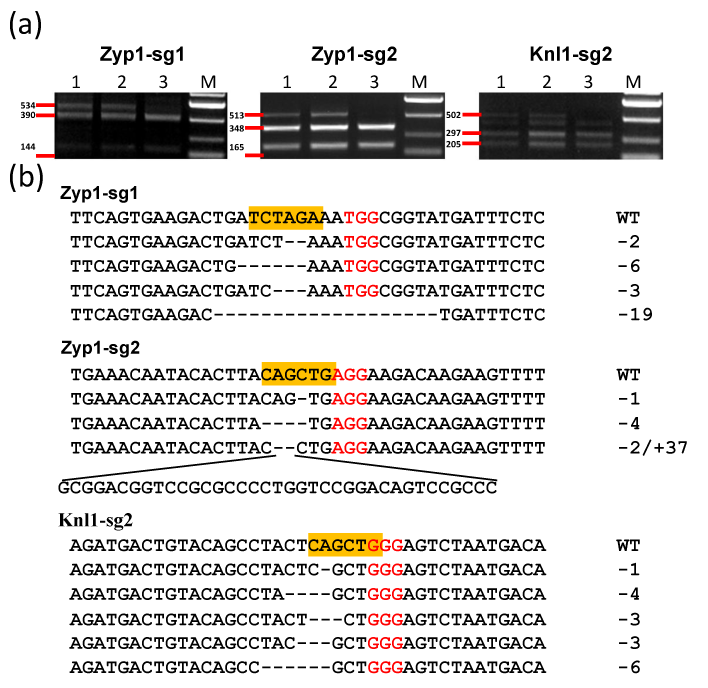


**Figure S5 Mutation analysis of the three sites targeted by DPC CRISPR/Cas9 system in protoplasts.**

(a) PCR-RE results of the three sites targeted. Two sites were located in the *zyp1* gene, named *zyp1*-sg1 and *zyp*-sg2; another site is located in the *knl1* gene, with the name *knl1*-sg2. For all the three target sites, lane 1 is sgRNAs co-transformed with *35S*-Cas9-SK (Cas9 driven by *35S* promoter, positive control) vector, lane 2 is sgRNAs co-transformed with *Dmc1*-Cas9-SK (Cas9 driven by *dmc1* promoter) vector, lane 3 is as negative control without plasmids transformed. M, DNA marker. (b) Mutations corresponding to lane 2 were confirmed by sequencing for each target site. Nucleotides marked in red represent PAM. The yellow box indicates the restriction site.


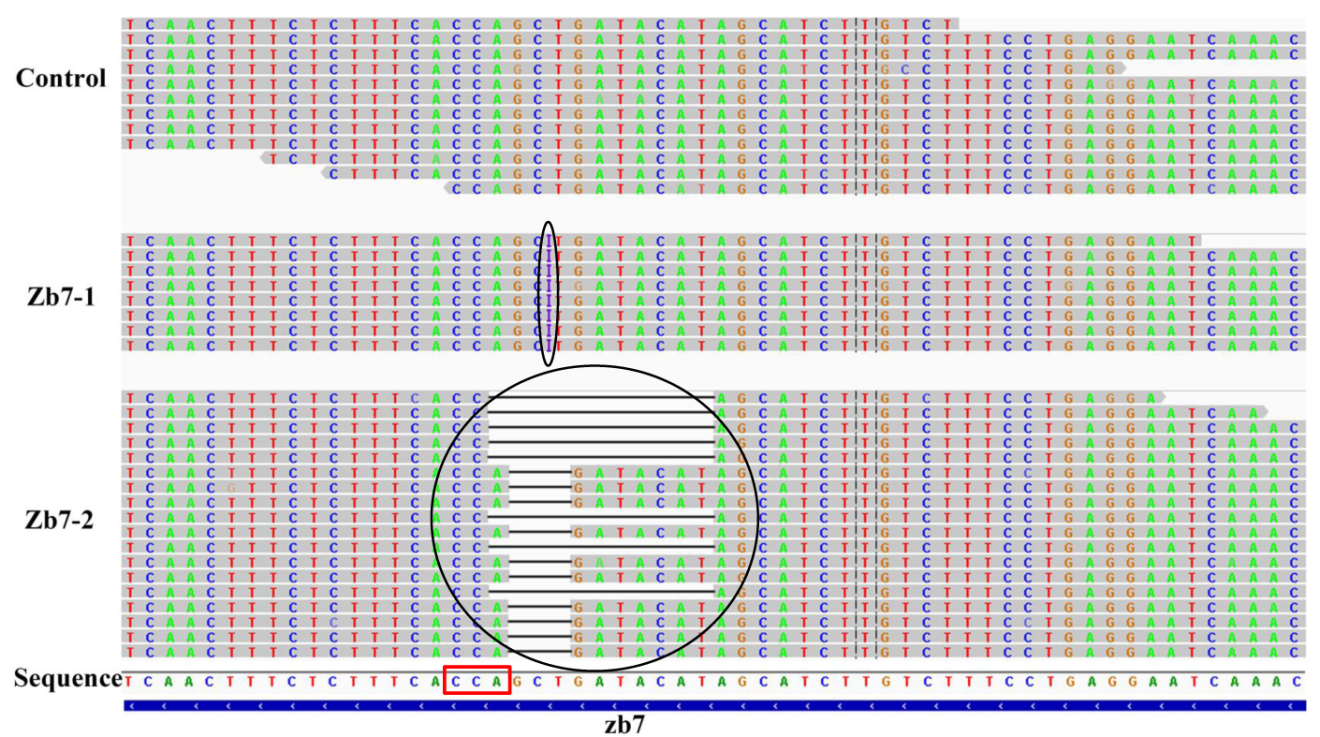


**Figure S6 Integrative Genomics Viewer (IGV) snapshots of the target site in the *zb7* gene.**

Two bi-allelic *zb7* mutants and one control were sampled for whole-genome re-sequencing and later analysis. The mutation regions are circled with black lines and the PAM regions are marked with red box. Insertions and deletions are shown using purple “ɫ” and black “-”, respectively.

gaattcgtaatcatgtcatagctgtttcctgtgtgaaattgttatccgctcacaattccacacaacatacgagccggaagcataaagtgtaaagcctggggtgcctaatgagtgagctaactcacattaattgcgttgcgctcactgcccgctttccagtcgggaaacctgtcgtgccagctgcattaatgaatcggccaacgcgcggggagaggcggtttgcgtattggagcttgagcttggatcagattgtcgtttcccgccttcagtttaaactatcagtgtttgacaggatatattggcgggtaaacctaagagaaaagagcgtttattagaataatcggatatttaaaagggcgtgaaaaggtttatccgttcgtccatttgtatgtgcatgccaaccacagggttcccctcgggatcaaagtactttaaagtactttaaagtactttaaagtactttgatccaacccctccgctgctatagtgcagtcggcttctgacgttcagtgcagccgtcttctgaaaacgacatgtcgcacaagtcctaagttacgcgacaggctgccgccctgcccttttcctggcgttttcttgtcgcgtgttttagtcgcataaagtagaatacttgcgactagaaccggagacattacgccatgaacaagagcgccgccgctggcctgctgggctatgcccgcgtcagcaccgacgaccaggacttgaccaaccaacgggccgaactgcacgcggccggctgcaccaagctgttttccgagaagatcaccggcaccaggcgcgaccgcccggagctggccaggatgcttgaccacctacgccctggcgacgttgtgacagtgaccaggctagaccgcctggcccgcagcacccgcgacctactggacattgccgagcgcatccaggaggccggcgcgggcctgcgtagcctggcagagccgtgggccgacaccaccacgccggccggccgcatggtgttgaccgtgttcgccggcattgccgagttcgagcgttccctaatcatcgaccgcacccggagcgggcgcgaggccgccaaggcccgaggcgtgaagtttggcccccgccctaccctcaccccggcacagatcgcgcacgcccgcgagctgatcgaccaggaaggccgcaccgtgaaagaggcggctgcactgcttggcgtgcatcgctcgaccctgtaccgcgcacttgagcgcagcgaggaagtgacgcccaccgaggccaggcggcgcggtgccttccgtgaggacgcattgaccgaggccgacgccctggcggccgccgagaatgaacgccaagaggaacaagcatgaaaccgcaccaggacggccaggacgaaccgtttttcattaccgaagagatcgaggcggagatgatcgcggccgggtacgtgttcgagccgcccgcgcacgtctcaaccgtgcggctgcatgaaatcctggccggtttgtctgatgccaagctggcggcctggccggccagcttggccgctgaagaaaccgagcgccgccgtctaaaaaggtgatgtgtatttgagtaaaacagcttgcgtcatgcggtcgctgcgtatatgatgcgatgagtaaataaacaaatacgcaaggggaacgcatgaaggttatcgctgtacttaaccagaaaggcgggtcaggcaagacgaccatcgcaacccatctagcccgcgccctgcaactcgccggggccgatgttctgttagtcgattccgatccccagggcagtgcccgcgattgggcggccgtgcgggaagatcaaccgctaaccgttgtcggcatcgaccgcccgacgattgaccgcgacgtgaaggccatcggccggcgcgacttcgtagtgatcgacggagcgccccaggcggcggacttggctgtgtccgcgatcaaggcagccgacttcgtgctgattccggtgcagccaagcccttacgacatatgggccaccgccgacctggtggagctggttaagcagcgcattgaggtcacggatggaaggctacaagcggcctttgtcgtgtcgcgggcgatcaaaggcacgcgcatcggcggtgaggttgccgaggcgctggccgggtacgagctgcccattcttgagtcccgtatcacgcagcgcgtgagctacccaggcactgccgccgccggcacaaccgttcttgaatcagaacccgagggcgacgctgcccgcgaggtccaggcgctggccgctgaaattaaatcaaaactcatttgagttaatgaggtaaagagaaaatgagcaaaagcacaaacacgctaagtgccggccgtccgagcgcacgcagcagcaaggctgcaacgttggccagcctggcagacacgccagccatgaagcgggtcaactttcagttgccggcggaggatcacaccaagctgaagatgtacgcggtacgccaaggcaagaccattaccgagctgctatctgaatacatcgcgcagctaccagagtaaatgagcaaatgaataaatgagtagatgaattttagcggctaaaggaggcggcatggaaaatcaagaacaaccaggcaccgacgccgtggaatgccccatgtgtggaggaacgggcggttggccaggcgtaagcggctgggttgtctgccggccctgcaatggcactggaacccccaagcccgaggaatcggcgtgagcggtcgcaaaccatccggcccggtacaaatcggcgcggcgctgggtgatgacctggtggagaagttgaaggccgcgcaggccgcccagcggcaacgcatcgaggcagaagcacgccccggtgaatcgtggcaagcggccgctgatcgaatccgcaaagaatcccggcaaccgccggcagccggtgcgccgtcgattaggaagccgcccaagggcgacgagcaaccagattttttcgttccgatgctctatgacgtgggcacccgcgatagtcgcagcatcatggacgtggccgttttccgtctgtcgaagcgtgaccgacgagctggcgaggtgatccgctacgagcttccagacgggcacgtagaggtttccgcagggccggccggcatggccagtgtgtgggattacgacctggtactgatggcggtttcccatctaaccgaatccatgaaccgataccgggaagggaagggagacaagcccggccgcgtgttccgtccacacgttgcggacgtactcaagttctgccggcgagccgatggcggaaagcagaaagacgacctggtagaaacctgcattcggttaaacaccacgcacgttgccatgcagcgtacgaagaaggccaagaacggccgcctggtgacggtatccgagggtgaagccttgattagccgctacaagatcgtaaagagcgaaaccgggcggccggagtacatcgagatcgagctagctgattggatgtaccgcgagatcacagaaggcaagaacccggacgtgctgacggttcaccccgattactttttgatcgatcccggcatcggccgttttctctaccgcctggcacgccgcgccgcaggcaaggcagaagccagatggttgttcaagacgatctacgaacgcagtggcagcgccggagagttcaagaagttctgtttcaccgtgcgcaagctgatcgggtcaaatgacctgccggagtacgatttgaaggaggaggcggggcaggctggcccgatcctagtcatgcgctaccgcaacctgatcgagggcgaagcatccgccggttcctaatgtacggagcagatgctagggcaaattgccctagcaggggaaaaaggtcgaaaaggtctctttcctgtggatagcacgtacattgggaacccaaagccgtacattgggaaccggaacccgtacattgggaacccaaagccgtacattgggaaccggtcacacatgtaagtgactgatataaaagagaaaaaaggcgatttttccgcctaaaactctttaaaacttattaaaactcttaaaacccgcctggcctgtgcataactgtctggccagcgcacagccgaagagctgcaaaaagcgcctacccttcggtcgctgcgctccctacgccccgccgcttcgcgtcggcctatcgcggccgctggccgctcaaaaatggctggcctacggccaggcaatctaccagggcgcggacaagccgcgccgtcgccactcgaccgccggcgcccacatcaaggcaccctgcctcgcgcgtttcggtgatgacggtgaaaacctctgacacatgcagctcccggagacggtcacagcttgtctgtaagcggatgccgggagcagacaagcccgtcagggcgcgtcagcgggtgttggcgggtgtcggggcgcagccatgacccagtcacgtagcgatagcggagtgtatactggcttaactatgcggcatcagagcagattgtactgagagtgcaccatatgcggtgtgaaataccgcacagatgcgtaaggagaaaataccgcatcaggcgctcttccgcttcctcgctcactgactcgctgcgctcggtcgttcggctgcggcgagcggtatcagctcactcaaaggcggtaatacggttatccacagaatcaggggataacgcaggaaagaacatgtgagcaaaaggccagcaaaaggccaggaaccgtaaaaaggccgcgttgctggcgtttttccataggctccgcccccctgacgagcatcacaaaaatcgacgctcaagtcagaggtggcgaaacccgacaggactataaagataccaggcgtttccccctggaagctccctcgtgcgctctcctgttccgaccctgccgcttaccggatacctgtccgcctttctcccttcgggaagcgtggcgctttctcatagctcacgctgtaggtatctcagttcggtgtaggtcgttcgctccaagctgggctgtgtgcacgaaccccccgttcagcccgaccgctgcgccttatccggtaactatcgtcttgagtccaacccggtaagacacgacttatcgccactggcagcagccactggtaacaggattagcagagcgaggtatgtaggcggtgctacagagttcttgaagtggtggcctaactacggctacactagaaggacagtatttggtatctgcgctctgctgaagccagttaccttcggaaaaagagttggtagctcttgatccggcaaacaaaccaccgctggtagcggtggtttttttgtttgcaagcagcagattacgcgcagaaaaaaaggatctcaagaagatcctttgatcttttctacggggtctgacgctcagtggaacgaaaactcacgttaagggattttggtcatgcatgatatatctcccaatttgtgtagggcttattatgcacgcttaaaaataataaaagcagacttgacctgatagtttggctgtgagcaattatgtgcttagtgcatctaacgcttgagttaagccgcgccgcgaagcggcgtcggcttgaacgaatttctagctagacattatttgccgactaccttggtgatctcgcctttcacgtagtggacaaattcttccaactgatctgcgcgcgaggccaagcgatcttcttcttgtccaagataagcctgtctagcttcaagtatgacgggctgatactgggccggcaggcgctccattgcccagtcggcagcgacatccttcggcgcgattttgccggttactgcgctgtaccaaatgcgggacaacgtaagcactacatttcgctcatcgccagcccagtcgggcggcgagttccatagcgttaaggtttcatttagcgcctcaaatagatcctgttcaggaaccggatcaaagagttcctccgccgctggacctaccaaggcaacgctatgttctcttgcttttgtcagcaagatagccagatcaatgtcgatcgtggctggctcgaagatacctgcaagaatgtcattgcgctgccattctccaaattgcagttcgcgcttagctggataacgccacggaatgatgtcgtcgtgcacaacaatggtgacttctacagcgcggagaatctcgctctctccaggggaagccgaagtttccaaaaggtcgttgatcaaagctcgccgcgttgtttcatcaagccttacggtcaccgtaaccagcaaatcaatatcactgtgtggcttcaggccgccatccactgcggagccgtacaaatgtacggccagcaacgtcggttcgagatggcgctcgatgacgccaactacctctgatagttgagtcgatacttcggcgatcaccgcttcccccatgatgtttaactttgttttagggcgactgccctgctgcgtaacatcgttgctgctccataacatcaaacatcgacccacggcgtaacgcgcttgctgcttggatgcccgaggcatagactgtaccccaaaaaaacagtcataacaagccatgaaaaccgccactgcgccgttaccaccgctgcgttcggtcaaggttctggaccagttgcgtgacggcagttacgctacttgcattacagcttacgaaccgaacgaggcttatgtccactgggttcgtgcccgaattgatcacaggcagcaacgctctgtcatcgttacaatcaacatgctaccctccgcgagatcatccgtgtttcaaacccggcagcttagttgccgttcttccgaatagcatcggtaacatgagcaaagtctgccgccttacaacggctctcccgctgacgccgtcccggactgatgggctgcctgtatcgagtggtgattttgtgccgagctgccggtcggggagctgttggctggctggtggcaggatatattgtggtgtaaacaaattgacgcttagacaacttaataacacattgcggacgtttttaatgtactgaattaacgccgaattgctctagcattcgccattcaggctgcgcaactgttgggaagggcgatcggtgcgggcctcttcgctattacgccagctggcgaaagggggatgtgctgcaaggcgattaagttgggtaacgccagggttttcccagtcacgacgttgtaaaacgacggccagtgccaagctaattcgcttcaagacgtgctcaaatcactatttccacacccctatatttctattgcactcccttttaactgttttttattacaaaaatgccctggaaaatgcactccctttttgtgtttgtttttttgtgaaacgatgttgtcaggtaatttatttgtcagtctactatggtggcccattatattaatagcaactgtcggtccaatagacgacgtcgattttctgcatttgtttaaccacgtggattttatgacattttatattagttaatttgtaaaacctacccaattaaagacctcatatgttctaaagactaatacttaatgataacaattttcttttagtgaagaaagggataattagtaaatatggaacaagggcagaagatttattaaagccgcggtaagagacaacaagtaggtacgtggagtgtcttaggtgacttacccacataacataaagtgacattaacaaacatagctaatgctcctatttgaatagtgcatatcagcataccttattacatatagataggagcaaactctagctagattgttgagcagatctcggtgacgggcaggaccggacggggcggtaccggcaggctgaagtccagctgccagaaacccacgtcatgccagttcccgtgcttgaagccggccgcccgcagcatgccgcggggggcatatccgagcgcctcgtgcatgcgcacgctcgggtcgttgggcagcccgatgacagcgaccacgctcttgaagccctgtgcctccagggacttcagcaggtgggtgtagagcgtggagcccagtcccgtccgctggtggcggggggagacgtacacggtcgactcggccgtccagtcgtaggcgttgcgtgccttccaggggcccgcgtaggcgatgccggcgacctcgccgtccacctcggcgacgagccagggatagcgctcccgcagacggacgaggtcgtccgtccactcctgcggttcctgcggctcggtacggaagttgaccgtgcttgtctcgatgtagtggttgacgatggtgcagaccgccggcatgtccgcctcggtggcacggcggatgtcggccgggcgtcgttctgggctcatggtagatcccccgttcgtaaatggtgaaaattttcagaaaattgcttttgctttaaaagaaatgatttaaattgctgcaatagaagtagaatgcttgattgcttgagattcgtttgttttgtatatgttgtgttgagaattaattctcgaggtcctctccaaatgaaatgaacttccttatatagaggaagggtcttgcgaaggatagtgggattgtgcgtcatcccttacgtcagtggagatatcacatcaatccacttgctttgaagacgtggttggaacgtcttctttttccacgatgctcctcgtgggtgggggtccatctttgggaccactgtcggtagaggcatcttgaacgatagcctttcctttatcgcaatgatggcatttgtaggagccaccttccttttccactatcttcacaataaagtgacagatagctgggcaatggaatccgaggaggtttccggatattaccctttgttgaaaagtctcaattgccctttggtcttctgagactgtatctttgatatttttggagtagacaagtgtgtcgtgctccaccatgttatcacatcaatccacttgctttgaagacgtggttggaacgtcttctttttccacgatgctcctcgtgggtgggggtccatctttgggaccactgtcggcagaggcatcttcaacgatggcctttcctttatcgcaatgatggcatttgtaggagccaccttccttttccactatcttcacaataaagtgacagatagctgggcaatggaatccgaggaggtttccggatattaccctttgttgaaaagtctcaattgccctttggtcttctgagactgtatctttgatatttttggagtagacaagtgtgtcgtgctccaccatgttgacctgcaggcatgcaagcttgcatgcctgcaggtcgactctagaggatccccgggttttcaaagcgcatcctctcgcaaaggagcaggaagtttttcagtatagaacaaaatcccctggactaaaccatccaggtatctcagcagttttccactggttttcattttcaaaaacagctactggacttcccgtccatcatagctcacgactaaaccaccggacctttttaaaaaccacttttcaaaagcacctttttttggaaaacaaaacactaattgtcataccacactagacttgtccattcctgtggacatggactattcgaataggttttcaaactctgcgtagaggtgtacactttacccactagtccggctctgcgatctcatggccaatgagacccgaaaccgaatctctttctttcctcgcacgtccttaccttaacggttatatcggaaggagtcaggccaccgccatgtccaaactagacaaaacattccccctccttatcctcccggtgctccccagccttcataaccctggggtttggaccgcacgagttcagattgagtgtctacccatacagtctcgagtggttgtacttatcatgagtacagttagtgaaggatgacaaaccagtccttatatgagaggacaatccttctgctcacacctaacctggctgagccaacacctgaggccctcccctaaaccagggagtccctgatgatccctactcaaaggtgataagggtgaaaacccttcattatacacattttgaaaagcattttcttttgatcccggctgggtggccataataacttgtctcaaaatcatatcatgcataaaataacaggctgagggttgtggttgaaaaatcataggtaatttatgcatcaaagggatccagtgagtttgtcgtgcttatccgacgaagggggaagaggagctcgcggaactgtcttctagctccactgcctggcgtagacttgcagatctggcctccacgagacgacatgaacgctccgataactatgcaacatgaataagcaaacatacaaaccaacaagtataccaacaaatatttagtatagtggtcagaatagcgatatatggatgggtagagtcgtgagtagaatctgtgtcatgtggtgttgagatactactagtggtggagcggaggtgcttaccaggagggtggactggaggcgaagcgacactatgcgtgtagccggcagagcggagtaactaagtgagtggggtgtttgccttggctgagggtgttgagtgtgtgtggagagggagagggtagctgggtgtatttatagctgggtgtatgtggtgtagcacaatgaagttcattgttggtgagaatagtgacatatgaatcgtctgacttagtatgaacaggagagttatatgcagaatatgggacaagagtattttgggagttttctggaatatgaaccatacttaagtgatgacatggttggatagggaataatttgataagaatttagaaacaagaattattggaatcggagtttggaagcctggttcgaaggaatctcaaagttaagcgtgctcaacttagagaaacctgggatgggtgaccagatgggaagttccctactggaaggaaaatcacagtcaccggagttcgtatgactgaaatatgggtctggctggtcttaggtggactggatagcatgatggatgagtagttgaaatttagggtgatcggatgatcgatgaatagtgtcttggaatgagaaggaagaaagcacgattgcaaaagctaagcgacaagagcgacaaataacacacagatcactctctctctcaagtcactaatcactaatgatcacttgtcttaattgtggaacttggagagattggaagctttgattgtgtcttggaatggattgctagctcttgtattgaatgtgaaggattggaatgcttgggtgtcatgaatggaggtggttggggttgtatttatagccctcaaccacttcctagccgttgctccttttctaccgaccgcggacggtccgcgcccctggtccggacagtccgcccctgcacatcaacggctgaaatcgcaacgatcagcagtaacggctatatcaacggctatatagcatttaatgtgtcgtcagatgtcagataaaagcagtcgcagacggtccggtcatgcaccccggacggtccgcgaggatgctataattcattttaccgaacccgtcaccttcgggtttttcggttcttcaccgacctgatggtccgcgcctgaggccggatggtccgcgcttggtctcggacggtgcttggctttccatcggacggtccgtagtgtagacttgtgtttttgtattggttctgtcctaggctcaccctagtttcgcggacggtccgccgcaagggcccagacggtccgcgcttatgtgattttccaaaaagcttctcctgtccagaataatctacggtattccggacagtcgactttagaatagttgtagatgaacttatgcacctgtggaatgatcaattagacaaaccggttagtccacaaggtttgtgatggtcgtcaaacaccaaaaccgattatagggaatattgaaactatttccctttcaattatatttgtttagttgaggttctaaattttttatagcaaggcccgtttggttagagagactaattttagtccctgacttttagtctcatttagtctctattttgccaaacggaaggactaaagtagggactaattggttttagggcatgtttggttcgttacctcaattgccacattttgcctaacttttctgcctaaggttagttattcaattcgaataactaaccttaggcaaagtggggcacagttagccacaaaccaaacaagcccttagtctttagtcccttacatagatgctaaaagggactaaaggggaatatttactctaattacccttgcctagaaaactagtgtgaaacaaaaaaaagagtattttgatctttatgtattacatttaatgtatttaaaatctgtttagcccctacaactaaacaatatagagactaaagtttagtttagggactaaactttagtcctaagactaatggagccaaacggggcccaaatctcgtttaaaaatttgaagcaaacacatccttaacggaccgtgggcaatgagctgagtctcccctgactctgggccgcaaagtcctggtacacttgatcatgttggtccatcctatggctgaccgcgacttccctagactgaaatcaacccattcctaggctcctagcccagcccaacggccgagcacacgatccgttcgagagcgagaaggtcctccggcctcagcaccctcaagtacacagtacaccctcgcaccggtaccgcggtccgcggcacacgacacccccactcgtctgtcgactcacgtctgatcgtctctccccaacaatctctcgagtcttgccattcgcctcagcttgcccctcctccaagcgtccaagccccacccggccattgcctcctcctcctgccgcaggtaagctagctgctccagcttctcttgccatcgcgtgtcctgcactcaccgccctcgcgcgtgtaactcctcctccgtcccccgatcgggctactagtgcaggcacatggactataaggaccacgacggagactacaaggatcatgatattgattacaaagacgatgacgataagatggccccaaagaagaagcggaaggtcggtatccacggagtcccagcagccgacaagaagtacagcatcggcctggacatcggcaccaactctgtgggctgggccgtgatcaccgacgagtacaaggtgcccagcaagaaattcaaggtgctgggcaacaccgaccggcacagcatcaagaagaacctgatcggagccctgctgttcgacagcggcgaaacagccgaggccacccggctgaagagaaccgccagaagaagatacaccagacggaagaaccggatctgctatctgcaagagatcttcagcaacgagatggccaaggtggacgacagcttcttccacagactggaagagtccttcctggtggaagaggataagaagcacgagcggcaccccatcttcggcaacatcgtggacgaggtggcctaccacgagaagtaccccaccatctaccacctgagaaagaaactggtggacagcaccgacaaggccgacctgcggctgatctatctggccctggcccacatgatcaagttccggggccacttcctgatcgagggcgacctgaaccccgacaacagcgacgtggacaagctgttcatccagctggtgcagacctacaaccagctgttcgaggaaaaccccatcaacgccagcggcgtggacgccaaggccatcctgtctgccagactgagcaagagcagacggctggaaaatctgatcgcccagctgcccggcgagaagaagaatggcctgttcggaaacctgattgccctgagcctgggcctgacccccaacttcaagagcaacttcgacctggccgaggatgccaaactgcagctgagcaaggacacctacgacgacgacctggacaacctgctggcccagatcggcgaccagtacgccgacctgtttctggccgccaagaacctgtccgacgccatcctgctgagcgacatcctgagagtgaacaccgagatcaccaaggcccccctgagcgcctctatgatcaagagatacgacgagcaccaccaggacctgaccctgctgaaagctctcgtgcggcagcagctgcctgagaagtacaaagagattttcttcgaccagagcaagaacggctacgccggctacattgacggcggagccagccaggaagagttctacaagttcatcaagcccatcctggaaaagatggacggcaccgaggaactgctcgtgaagctgaacagagaggacctgctgcggaagcagcggaccttcgacaacggcagcatcccccaccagatccacctgggagagctgcacgccattctgcggcggcaggaagatttttacccattcctgaaggacaaccgggaaaagatcgagaagatcctgaccttccgcatcccctactacgtgggccctctggccaggggaaacagcagattcgcctggatgaccagaaagagcgaggaaaccatcaccccctggaacttcgaggaagtggtggacaagggcgcttccgcccagagcttcatcgagcggatgaccaacttcgataagaacctgcccaacgagaaggtgctgcccaagcacagcctgctgtacgagtacttcaccgtgtataacgagctgaccaaagtgaaatacgtgaccgagggaatgagaaagcccgccttcctgagcggcgagcagaaaaaggccatcgtggacctgctgttcaagaccaaccggaaagtgaccgtgaagcagctgaaagaggactacttcaagaaaatcgagtgcttcgactccgtggaaatctccggcgtggaagatcggttcaacgcctccctgggcacataccacgatctgctgaaaattatcaaggacaaggacttcctggacaatgaggaaaacgaggacattctggaagatatcgtgctgaccctgacactgtttgaggacagagagatgatcgaggaacggctgaaaacctatgcccacctgttcgacgacaaagtgatgaagcagctgaagcggcggagatacaccggctggggcaggctgagccggaagctgatcaacggcatccgggacaagcagtccggcaagacaatcctggatttcctgaagtccgacggcttcgccaacagaaacttcatgcagctgatccacgacgacagcctgacctttaaagaggacatccagaaagcccaggtgtccggccagggcgatagcctgcacgagcacattgccaatctggccggcagccccgccattaagaagggcatcctgcagacagtgaaggtggtggacgagctcgtgaaagtgatgggccggcacaagcccgagaacatcgtgatcgaaatggccagagagaaccagaccacccagaagggacagaagaacagccgcgagagaatgaagcggatcgaagagggcatcaaagagctgggcagccagatcctgaaagaacaccccgtggaaaacacccagctgcagaacgagaagctgtacctgtactacctgcagaatgggcgggatatgtacgtggaccaggaactggacatcaaccggctgtccgactacgatgtggaccatatcgtgcctcagagctttctgaaggacgactccatcgacaacaaggtgctgaccagaagcgacaagaaccggggcaagagcgacaacgtgccctccgaagaggtcgtgaagaagatgaagaactactggcggcagctgctgaacgccaagctgattacccagagaaagttcgacaatctgaccaaggccgagagaggcggcctgagcgaactggataaggccggcttcatcaagagacagctggtggaaacccggcagatcacaaagcacgtggcacagatcctggactcccggatgaacactaagtacgacgagaatgacaagctgatccgggaagtgaaagtgatcaccctgaagtccaagctggtgtccgatttccggaaggatttccagttttacaaagtgcgcgagatcaacaactaccaccacgcccacgacgcctacctgaacgccgtcgtgggaaccgccctgatcaaaaagtaccctaagctggaaagcgagttcgtgtacggcgactacaaggtgtacgacgtgcggaagatgatcgccaagagcgagcaggaaatcggcaaggctaccgccaagtacttcttctacagcaacatcatgaactttttcaagaccgagattaccctggccaacggcgagatccggaagcggcctctgatcgagacaaacggcgaaaccggggagatcgtgtgggataagggccgggattttgccaccgtgcggaaagtgctgagcatgccccaagtgaatatcgtgaaaaagaccgaggtgcagacaggcggcttcagcaaagagtctatcctgcccaagaggaacagcgataagctgatcgccagaaagaaggactgggaccctaagaagtacggcggcttcgacagccccaccgtggcctattctgtgctggtggtggccaaagtggaaaagggcaagtccaagaaactgaagagtgtgaaagagctgctggggatcaccatcatggaaagaagcagcttcgagaagaatcccatcgactttctggaagccaagggctacaaagaagtgaaaaaggacctgatcatcaagctgcctaagtactccctgttcgagctggaaaacggccggaagagaatgctggcctctgccggcgaactgcagaagggaaacgaactggccctgccctccaaatatgtgaacttcctgtacctggccagccactatgagaagctgaagggctcccccgaggataatgagcagaaacagctgtttgtggaacagcacaagcactacctggacgagatcatcgagcagatcagcgagttctccaagagagtgatcctggccgacgctaatctggacaaagtgctgtccgcctacaacaagcaccgggataagcccatcagagagcaggccgagaatatcatccacctgtttaccctgaccaatctgggagcccctgccgccttcaagtactttgacaccaccatcgaccggaagaggtacaccagcaccaaagaggtgctggacgccaccctgatccaccagagcatcaccggcctgtacgagacacggatcgacctgtctcagctgggaggcgacaaaaggccggcggccacgaaaaaggccggccaggcaaaaaagaaaaagtaaggatcctgattgatcgatagagctcgaatttccccgatcgttcaaacatttggcaataaagtttcttaagattgaatcctgttgccggtcttgcgatgattatcatataatttctgttgaattacgttaagcatgtaataattaacatgtaatgcatgacgttatttatgagatgggtttttatgattagagtcccgcaattatacatttaatacgcgatagaaaacaaaatatagcgcgcaaactaggataaattatcgcgcgcggtgtcatctatgttactagatcgg

**Figure S7 DNA sequence of the p*Dmc1*-Cas9 binary vector**

Sequence marked in gray, green, pink and red represent pTF101.1 backbone, dmc1 promoter, hspCas9 and Nos terminator, respectively.
