# Supplementary material for: High‐efficiency genome editing using a dmc1 promoter‐controlled CRISPR/Cas9 system in maize
Source: Plant Biotechnol J. 2018 Apr 30;16(11):1848–57. doi: 10.1111/pbi.12920 (PMC6181213; doi:10.1111/pbi.12920)
Supplement: Supplementary file 2 — Table S1 Phenotype statistics of transgenic T0 plants targeted by DPC CRISPR/Cas9 system. Table S2 Genotyping of the T0 plants by direct‐sequencing of the PCR products. Table S3 Genotype statistics of transgenic T0 plants targeted by DPC CRISPR/Cas9 system at zyp1 gene. Table S4 Genotyping of the T1 plants by RFLP assay and sanger sequencing at the zb7 gene. Table S5 Summary of the re‐sequencing quality of different maize transgenic lines. Table S6 Summary of the putative off‐target examination. Table S7 Summary of the mutations of the whole genome re‐sequencing of maize transgenic lines and control. Table S8 Predicted CRISPR/Cas9 off‐target sites of the two gene zyp1 and zb7. Table S9 Maize genes targeted in this study. Table S10 Primers used in this study. [file PBI-16-1848-s001.docx]

**Table S1 Phenotype statistics of transgenic T0 plants targeted by DPC CRISPR/Cas9 system**

| **Transgenic events** | **Albino plants (%)** | **Chlorosis plants (%)** | **Chimeras (%)** | **Wildtype plants (%)** | | **Total** |
| --- | --- | --- | --- | --- | --- | --- |
| #1 | 29 (74) | 0 | 6 (54) | 4 | 39 | |
| #2 | 1 (5) | 0 | 1 (5) | 18 | 20 | |
| #3 | 41 (100) | 0 | 0 | 0 | 41 | |
| #4 | 18 (86) | 0 | 2 (9) | 1 (5) | 21 | |
| #5 | 9 (100) | 0 | 0 | 0 | 9 | |
| #6 | 0 | 16 (89) | 0 | 2 (11) | 18 | |
| #7 | 0 | 19 (100) | 0 | 0 | 19 | |
| #8 | 1 (7) | 0 | 10 (71) | 3 (22) | 14 | |
| #9 | 16 (57) | 0 | 11 (39) | 1 (4) | 28 | |
| #10 | 6 (21) | 0 | 15 (54) | 7 (25) | 28 | |
| Total | 121 (51) | 35 (15) | 45(19) | 36(15) | 237 | |

**Table S2 Genotyping of the T0 plants by direct-sequencing of the PCR products**

| **Event**^†^ **#1** | **Zygosity**^‡^ | **Genotype** | **Event #2** | **Zygosity** | **Genotype** | **Event #3** | **Zygosity** | **Genotype** | **Event #4** | **Zygosity** | **Genotype** |
| --- | --- | --- | --- | --- | --- | --- | --- | --- | --- | --- | --- |
| 1-1 | Homozygote | i1/i1 | 2-1 | Chimera | ND | 3-1 | Bi-allele | i1/d1 | 4-1 | Bi-allele | d2/d1 |
| 1-2 | Homozygote | i1/i1 | 2-2 | Chimera | ND | 3-2 | Bi-allele | i1/d1 | 4-2 | Bi-allele | i1a/i1b |
| 1-3 | Homozygote | i1/i1 | 2-3 | Chimera | ND | 3-3 | Bi-allele | i1/d1 | 4-3 | Homozygote | i1/i1 |
| 1-4 | Bi-allele | i1/d29 | 2-4 | Chimera | ND | 3-4 | Bi-allele | i1/d1 | 4-4 | Homozygote | i1/i1 |
| 1-5 | Homozygote | i1/i1 | 2-5 | Chimera | ND | 3-5 | Bi-allele | i1/d1 | 4-5 | Homozygote | i1/i1 |
| 1-6 | Homozygote | i1/i1 | 2-6 | Chimera | ND | 3-6 | Bi-allele | i1/d1 | 4-6 | Homozygote | i1/i1 |
| 1-7 | Homozygote | i1/i1 | 2-7 | Chimera | ND | 3-7 | Bi-allele | i1/d1 | 4-7 | Homozygote | i1/i1 |
| 1-8 | Homozygote | i1/i1 | 2-8 | Chimera | ND | 3-8 | Bi-allele | i1/d1 | 4-8 | Homozygote | i1/i1 |
| 1-9 | Homozygote | i1/i1 | 2-9 | Chimera | ND | 3-9 | Bi-allele | i1/d1 | 4-9 | Homozygote | i1/i1 |
| 1-10 | Bi-allele | i1a/i1b | 2-10 | Chimera | ND | 3-10 | Bi-allele | i1/d1 | 4-10 | Bi-allele | i1/d2 |
| 1-11 | heterozygote | i1/WT | 2-11 | heterozygote | i1/WT |  |  |  | 4-11 | Chimera | ND |
| 1-12 | heterozygote | i1/WT | 2-12 | Chimera | ND |  |  |  |  |  |  |

†As more than one plants can be regenerated by one HiII transgenic callus, this one callus usually defined as one events, all seedlings regenerated from this callus belongs to same transgenic event.

‡The genotype is determined by RFLP assay (see Figure S2) and Sanger sequencing. d#, # of bp deleted at the target site. d#a and d#b, same number of deletion but different types at target sites. i#, # of bp insertion at target site; i#a and i#b, same number of insertion of different nucleotide at target site. c#, combined mutation.

**Table S3 Genotype statistics of transgenic T0 plants targeted by DPC CRISPR/Cas9 system at *zyp1* gene**

| **Transgene plant no.** | **Cas9** | **Zygosity** | **Genotype** | **Transgene plant no.** | **Cas9** | **Zygosity** | **Genotype** |
| --- | --- | --- | --- | --- | --- | --- | --- |
| 1 | + | Bi-allelic | i1/i2 | 14 | + | Bi-allelic | d3/(d8/i1) |
| 2 | + | homozygous | d2/d2 | 15 | + | Bi-allelic | i1a/i1b |
| 3 | + | Bi-allelic | d3/(d8/i1) | 16 | + | Bi-allelic | i1a/i1b |
| 4 | + | Bi-allelic | d3/(d8/i1) | 17 | + | Bi-allelic | i1a/i1b |
| 5 | + | Bi-allelic | i1/i2 | 18 | + | Bi-allelic | d1/d10 |
| 6 | + | Bi-allelic | d3/(d8/i1) | 19 | + | homozygous | i1/i1 |
| 7 | + | Bi-allelic | i1/d1 | 20 | + | Bi-allelic | i1/d1 |
| 8 | + | Bi-allelic | i1/d14 | 21 | + | Bi-allelic | d1/d10 |
| 9 | + | Bi-allelic | i1/d14 | 22 | + | Bi-allelic | d1/d10 |
| 10 | + | Bi-allelic | d2/(d4/i1) | 23 | + | Bi-allelic | d2/d8 |
| 11 | + | Bi-allelic | d2/(d4/i1) | 24 | + | heterozygous | d3/WT |
| 12 | + | Bi-allelic | i1/d3 | 25 | + | chimeric | N.D. |
| 13 | + | Bi-allelic | d3/(d8/i1) | 26 | + | chimeric | N.D. |

See table S2 for genotype denotations.

Table S4 Genotyping of the T1 plants by RFLP assay and sanger sequencing at the *zb7* gene

| **Cross (female X male)** | **T0** | | **T1** | | | |
| --- | --- | --- | --- | --- | --- | --- |
|  | **Female**  **genotype** | **Male**  **genotype** | **Genotype** | **Total plants** | **Cas9 free** | **Cas9-free plant genotype** |
| 1 10-16X10-23) | D3, WT^†^ | i1, WT | 11d3,i1 : 17d3,WT : 9i1,WT : 22WT,WT : 3d1,d3 : 2d1,i1 : 2d1,WT : 1d3,d3 : 1i1,i1 : 1d3,c3 : 1d2,WT | 70 | 3 | 2d3,WT : 1WTWT |
| 2 (8-1X10-14) | i1, WT | WT, WT | 4i1,i1 : 13i1,WT : 6WT,WT : 1i1,d2 : 1i1,d4 : 1i1,d7 : 1d1,d2 : 1d1,WT : 1d2,WT : 1d4,WT : 1d26,WT | 31 | 4 | 1i1,WT : 1d1,WT : 2WT,WT |
| 3 (9-2X10-28) | i1, WT | WT, WT | 1i1,i1 :6i1,WT : 2WT,WT : 1i1,d1 : 1i1,d2 : 1i1,d30 : 1d2,WT | 13 | 3 | 1i1,WT : 1i1,i1 : 1d1,i1 |
| 4 (10-1X10-14) | WT, WT | WT, WT | 12WT,WT : 4i1a,WT : 3d1,WT : 1d2,WT : 1i1b,WT : 1i1a,i1a : 1d2,c1 : 1d3,d3 | 24 | 1 | 1WTWT |
| 5 (10-3X10-28) | WT, WT | WT, WT | 3WT,WT : 1i1,i1 : 1i1,WT : 1d1,WT | 6 | 1 | 1i1,WT |

See table S2 for genotype denotations.

^†^The genotype “WT” is determined by Sanger sequencing results. Plants with allele “WT” may be mosaic plant.

**Table S5. Summary of the re-sequencing quality of different maize transgenic lines.**

| **Plant ID** | **Total # of reads** | **# of mapped reads** | **Mapping ratio** | **Average depth** |
| --- | --- | --- | --- | --- |
| zb7-1 | 124731276 | 123057724 | 98.66% | 14.79 |
| zb7-2 | 100720276 | 100229933 | 99.51% | 11.95 |
| control | 96561460 | 95833875 | 99.25% | 11.46 |

Control, regenerated Hi-II seedling sample without Cas9 transgene.

**Table S6. Summary of the putative off-target examination**

| **Plant ID** | **Seed sequence** | **# of putative targets** | **# of off-target site detected** |
| --- | --- | --- | --- |
| control | CAAGATGCTATGTATCAGCNGG | 1035 | 0 |
| zb7-1 |  |  | 0 |
| zb7-2 |  |  | 0 |

Control, regenerated Hi-II seedling sample without Cas9 transgene. Nucleotides marked in blue represent PAM.

**Table S7. Summary of the mutations of the whole genome re-sequencing of maize transgenic lines and control**

| **Plant ID** | **# of SNPs** | **# of insertions** | **# of deletions** | **Total # of mutations** |
| --- | --- | --- | --- | --- |
| zb7-1 | 8302593 | 441170 | 473977 | 9217819 |
| zb7-2 | 7863182 | 400756 | 430003 | 8694001 |
| control | 5799591 | 323696 | 338385 | 6461729 |

Control, regenerated Hi-II seedling sample without Cas9 transgene.

**Table S8 Predicted CRISPR/Cas9 off-target sites of the two gene *zyp1* and *zb7***

| **Target gene** | **Name of predicted off-target sites** | **Chromosome** | **Sequence of the predicted off-target site** | **No. of**  **mismatching**  **bases** |
| --- | --- | --- | --- | --- |
| *zyp1* | SG1 | chr10 | GTGAAGACTGATCTAGAAATGG | 0 |
|  | OT1 | chr3 | aTGAAGtCaGATCTAGAAATGG | 3 |
|  | OT2 | chr2 | GaGAAaACTGAaCTAGAAAAGG | 3 |
|  | OT3 | chr2 | GcGAAGgCTGtTCTAGAAAAGG | 3 |
|  | OT4 | chr6 | GTGAAGAaTGATCTAaAgACGG | 3 |
|  | OT5 | chr9 | GTGAAGACTGgaCaAGAAAAGG | 3 |
|  | OT6 | chr9 | aTaAAGACaGATCTAGAAAAGG | 3 |
| *zb7* | SG2 | chr1 | CAAGATGCTATGTATCAGCTGG | 0 |
|  | OT1 | chr7 | CcAGATGCTAaGTATCAGtTGG | 3 |

The red lowercase nucleotides indicate mismatches; nucleotides marked in blue represent PAM.

**Table S9. Maize genes targeted in this study**

| Target genes | Target sequences |
| --- | --- |
| *zb7* | CAAGATGCTATGTATCAGCTGG |
| *zyp1* (sg1) | GTGAAGACTGATCTAGAAATGG |
| *zyp1* (sg2) | AACAATACACTTACAGCTGAGG |
| *smc3* | GAACCTGCGAAGTGAAGATAGG |
| *knl1* | CTGTACAGCCTACTCAGCTGGG |

Nucleotides marked in blue represent PAM.

**Table S10 Primers used in this study**

| **Primer name** | **Primer sequence (5’-3’)** | **Description** |
| --- | --- | --- |
| dmcp-F | AATTGGGTACCGGGCCCCCCCCCGGGTTTTCAAAGCGCATCCTCTC | dmc1 promoter amplify |
| dmcp-R | TCGTGGTCCTTATAGTCCATGTGCCTGCACTAGTAGCCCGATC | dmc1 promoter amplify |
| sgRNA-F | AGGTCGACTCTAGAGGATCCGAATTCCATCTAAGTATCTTG | sgRNA sub-cloning |
| sgRNA-R | AGAGGATGCGCTTTGAAAACTGCAGAATTGCCCTTCGAAG | sgRNA sub-cloning |
| Cas9-F | ACCCCACCATCTACCACCTG | Transgene identification |
| Cas9-R | TGGGCAGCACCTTCTCGTTG | Transgene identification |
| zb7sg-F1 | AGCACAAGATGCTATGTATCAGC | Annealing for asembling into U3-sgRNA cassette |
| zb7sg-F2 | TTTTACAAGATGCTATGTATCAGC | Annealing for asembling into U6-sgRNA cassette |
| zb7sg-R | AAACGCTGATACATAGCATCTTG | Annealing for asembling into U3/U6-sgRNA cassette |
| zyp1sg-F1 | AGCAGTGAAGACTGATCTAGAAA | Annealing for asembling into U3-sgRNA cassette |
| zyp1sg-F2 | TTTTAAACAATACACTTACAGCTG | Annealing for asembling into U6-sgRNA cassette |
| zyp1sg-R | AAACTTTCTAGATCAGTCTTCAC | Annealing for asembling into U3/U6-sgRNA cassette |
| smc3sg-F | AGCAGAACCTGCGAAGTGAAGAT | Annealing for asembling into U3-sgRNA cassette |
| smc3sg-R | AAACATCTTCACTTCGCAGGTTC | Annealing for asembling into U3-sgRNA cassette |
| knl1sg-F | AGCACTGTACAGCCTACTCAGCT | Annealing for asembling into U3-sgRNA cassette |
| knl1sg-R | AAACAGCTGAGTAGGCTGTACAG | Annealing for asembling into U3-sgRNA cassette |
| zb7-F | CACTTCATGGCCTTCAATAC | Amplify the target site sequence |
| zb7-R | GCTGATCCTGTTTCCTGGTC | Amplify the target site sequence |
| zyp1-F | GCGAAACCGAGCACCCAAAC | Amplify the target site sequence |
| zyp1-R | CATGCTAGGGACAGGACAAC | Amplify the target site sequence |
| smc3-F | GGGCAATTACAACTTGGATG | Amplify the target site sequence |
| smc3-R | GGCAACCATCAGACCATCAC | Amplify the target site sequence |
| knl1-F | TTGCGTGTCCATGCTTCTAG | Amplify the target site sequence |
| knl1-R | TTTGCTTGCTCCCTTTTATC | Amplify the target site sequence |
| OT1-F | AAGTGGGTTTCATCCTCATC | Amplify the predicted off-target sequence |
| OT1-R | CGGGACAGGAATAGGTGAAG | Amplify the predicted off-target sequence |
| OT2-F | GCAGCACACCCAATACACAG | Amplify the predicted off-target sequence |
| OT2-R | TTCTCTCCCTGCTGGTCATG | Amplify the predicted off-target sequence |
| OT3-F | GCCTCCATATCCTCCTGTTG | Amplify the predicted off-target sequence |
| OT3-R | CATTGCCACTGGTCCCTTTC | Amplify the predicted off-target sequence |
| OT4-F | CTGCCCTGAGGAAAATGAAG | Amplify the predicted off-target sequence |
| OT4-R | TACGCTCTGAAGGCCCGAAC | Amplify the predicted off-target sequence |
| OT5-F | GGACGGCACGCTCGAAATAG | Amplify the predicted off-target sequence |
| OT5-R | CTGAATGGCCGGTCGGTTAC | Amplify the predicted off-target sequence |
| OT6-F | CGCTGGATGATGACCCTAAG | Amplify the predicted off-target sequence |
| OT6-R | CGATGCGGTTCTAATCCCAC | Amplify the predicted off-target sequence |
| OT7-F | GCGGGTTTAGGTGACCTTAC | Amplify the predicted off-target sequence |
| OT7-R | GCAAAACTTTCAGTCGATAG | Amplify the predicted off-target sequence |
